# Virtual, Nurse-Led Early Primary Palliative Care Intervention (ELICIT) for Community-Dwelling Older Adults With Cognitive Impairment: Protocol for a Randomized Controlled Trial

**DOI:** 10.2196/75082

**Published:** 2025-12-24

**Authors:** Vyjeyanthi S Periyakoil, Charles Von Gunten, Helena Kraemer

**Affiliations:** 1 Stanford University, 3180 Porter Dr, Palo Alto, CA, 94304, United States, 1 6507234000; 2 The Elizabeth Hospice, San Diego, CA, United States

**Keywords:** primary palliative care, goals of care, advance care planning, serious illness, supportive care, nursing, cognitive impairment, dementia, MCI

## Abstract

**Background:**

Although dementia is a serious illness that progresses over many years, little is known about the primary palliative care needs of individuals who have it, especially those living in the community.

**Objective:**

This trial aims to test the impact of a virtual, nurse-led early primary palliative care intervention (ELICIT) on older adults living in the community who are chronically ill and have a diagnosis of cognitive impairment or are at risk of it.

**Methods:**

A total of 200 community-dwelling older adults who were chronically ill and had varying degrees of cognitive impairment were recruited and randomized to either usual care or usual care + a virtual, nurse-led ELICIT. For both arms, we will track the number of participants who (1) report supportive care needs to the blinded evaluators and (2) complete conversations on goals of care and document advance directives and the Physician Orders for Life-Sustaining Treatment form in the electronic health record. We will also track their end-of-life resource use and the percentage of participants who receive goal-concordant care. Changes in Edmonton Symptom Assessment Scale, Patient Activation Measure, and Quality of Life in Alzheimer’s Disease scores will be tracked and analyzed.

**Results:**

As of October 2025, we have recruited 200 participants. We are following all study participants on an ongoing basis to determine whether they received goal-concordant care at the end of life and their resource use patterns. We hypothesize that, compared to the usual care arm, more participants in the intervention arm will (1) express supportive care needs to the blinded evaluators, (2) complete goals of care conversations, document advance care planning, and (3) have higher levels of goal-concordant care and lower end-of-life resource use.

**Conclusions:**

The identification of the primary palliative care needs of community-dwelling older adults who are chronically ill and have various levels of cognitive impairment will help refine the intervention and enable trained nurses to provide virtual early primary palliative care within the scope of nursing.

## Introduction

### Background

The World Health Organization has declared dementia a public health crisis and issued a call for global nations to make dementia an international public health priority [1]. Although dementia is a serious illness that progresses over many years, little is known about the everyday suffering endured over time by patients in the early stages of the disease, as most research focuses on patients who are already institutionalized and at the end of life. However, patients early in the dementia trajectory are beset with distressing symptoms. An estimated 85% [2] of persons with dementia live in the community (eg, private residences or non–nursing home residential care facilities) with unmet needs [3]. Proactive, patient-centered primary palliative care will likely benefit early-stage patients living in the community. Implementing early palliative care (EPC) will facilitate ongoing assessment and management of the distressing symptoms experienced by persons with dementia from very early in the illness trajectory, result in identification and management of primary palliative care needs, and facilitate timely advance care planning [4].

### Preliminary Studies Conducted in Shaping the Design of This Proposed Trial

#### Preliminary Study 1

Working with participants and families, we co-designed the What Matters Most letter advance directive (WMM) in a simple letter format written at a fifth-grade reading level in multiple languages (English, Chinese, Hindi, Russian, Spanish, Tagalog, Vietnamese, and Urdu). The WMM was then tested and validated through a large, randomized controlled trial (NCT02799537) [5]. Participants reported that this advance directive was easier to read, better reflected what mattered most to them, helped stimulate their thinking about the types of treatments they wanted at the end of life, and allowed them to describe how they made medical decisions in their family.

#### Preliminary Study 2

We demonstrated that the WMM facilitates patient-proxy collaboration in completing advance directives [6]. The participants and their proxies used the WMM to identify their points of agreement and disagreement about the participants’ choices for end-of-life care. When the participants and proxies collaborated in completing the participants’ WMM, they were able to identify their viewpoints and arrive at a consensus after discussing the items in the WMM. Notably, a structured participant-proxy discussion using the WMM helped them reconcile discordance, most often in favor of a participant’s original wishes. All participants and their proxies reported that they very much preferred the WMM to traditional advance directive documents.

Health care providers too preferred the WMM over traditional advance directives [6]. We conducted a prospective cluster randomized controlled trial with 5 general medicine ward teams at Stanford Hospital in 2016. The WMM was very helpful to the physicians, who were better able to understand their participants’ life goals and values and improve shared decision-making regarding goals of care and advance care planning. While this was important for all patients with chronic and serious illnesses, it was especially important for patients with cognitive impairment, who should complete advance care planning and provide anticipatory guidance while they still have the capacity to make informed decisions. These efforts are essential to ensure that their future care aligns with their values and goals.

#### Preliminary Study 3

To provide preference-sensitive care, it is important to routinely inquire about participants’ bucket lists and discuss the impact of their medical treatments on their life goals. We conducted a study with 3016 patients and identified 6 common themes: the desire to travel (78.5%), accomplish a personal goal (78.3%), achieve specific life milestones (51%), spend quality time with friends and family (16.7%), achieve financial stability (24.3%), and do a daring activity (15%) [7]. This study resulted in a simple life goal list tool that can be used to engage patients in their health care decision-making.

### Trial Objectives and Purpose

The aim of this study is to test the impact of a virtual, nurse-led early primary palliative care intervention (ELICIT) on older adults living in the community who are chronically ill and have a diagnosis of cognitive impairment or who are at risk of it. We hypothesize that, compared to the usual care (UC) arm, more participants in the intervention arm will (1) express supportive care needs to the blinded evaluators, (2) complete conversations on goals of care and advance care planning documentation, and (3) have lower end-of-life resource use (hospital admissions and days and emergency visits) and higher levels of goal-concordant care.

## Methods

### Study Design

#### Overview

**Figure 1.**
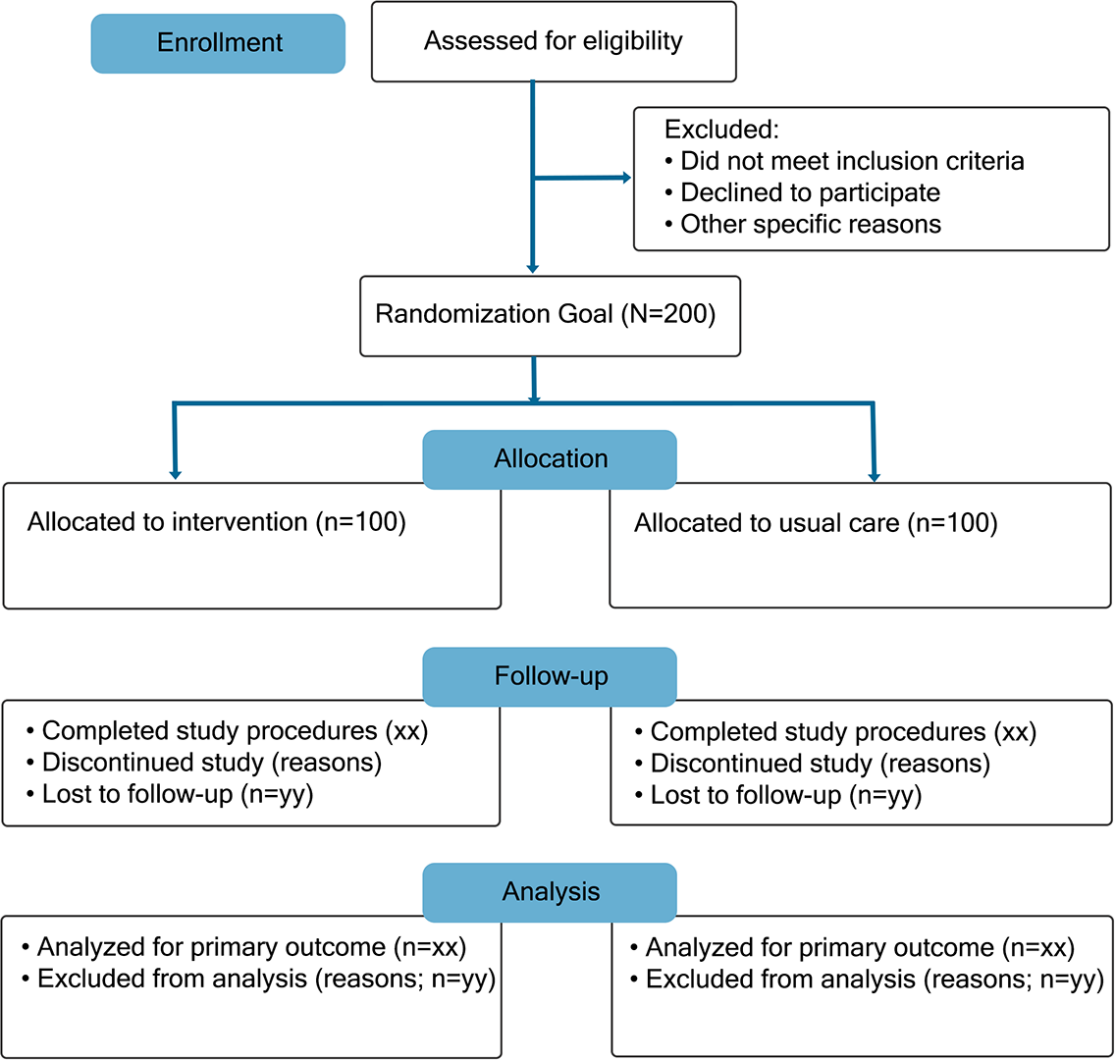
CONSORT (Consolidated Standards of Reporting Trials) diagram.

This study aimed to recruit 200 community-dwelling older adults (Figure 1) who were chronically ill and at risk of cognitive impairment or already had it and had undergone a full assessment by a multidisciplinary neurology team. They were randomized (100 participants in each arm) to UC or UC + a virtual EPC intervention delivered by a nurse over a 12-month period.

#### Impact of the COVID-19 Pandemic and Study Redesign

This study was originally designed to test the impact of an in-person nurse intervention to be delivered to the participants at their homes. Just as we were launching the study, the COVID-19 pandemic started in 2020, and the shelter-in-place orders were imposed. The study team had to adapt our study design to be able to proceed with the trial under the constraints imposed by COVID-19.

The following factors were key in shaping the study redesign:

1. All in-person research visits and face-to-face encounters were suspended due to the pandemic. Therefore, we had to change the study design to make it a virtual study.
2. In redesigning our study, we had to account for the technical challenges that our study participants (older adults) faced. In 2020, many older adults did not know how to participate in video encounters. We had to arrange for routine technical assistance before every encounter for participants who needed it.
3. We piloted the study survey outcome measures virtually. As the measures had Likert-scale responses with some reverse-worded items, it was challenging for the participants to respond over video or phone. On the basis of pilot participant feedback, the Likert measures became secondary outcome measures.
4. Originally, we wanted to observe the dyadic interactions between the participants and their care partners in person and track the caregivers’ Zarit Burden Interview scores [8] over time. However, many of the caregivers were sheltered in place due to COVID-19 and not able to live with the participants. Due to COVID-19, many care partners were not available, and we could not track dyadic interactions.

#### Final Study Design

We converted the in-person nurse intervention to a virtual intervention.

### Control Condition (UC)

Participants randomized to the UC arm received care from their primary care provider (PCP) and their care team. In this case, UC refers to the standard or routine outpatient care that participants received from their primary care team. This included any appointments, consultations, and procedures that were standard for outpatient primary care.

### Intervention Arm (EPC)

Participants in the intervention arm received UC + a virtual, nurse-led ELICIT.

### Training of Study Nurses to Conduct the Virtual Intervention

All study nurses were trained using a training program provided through a learning management system followed by immersive simulation training with standardized patients. The feedback of the standardized patients and the preceptor was used to refine their skills. Ongoing training for skill building and education was provided through nurse peer support, weekly meetings with the study physician, and the weekly trial team meetings.

### Four Components of Primary Palliative Care for the Nurse-Led Intervention

The nurse-led intervention was designed to focus on the four key components of primary palliative care: (1) assessing and palliating distressing physical symptoms; (2) assessing and supporting psychological, social, cultural, and spiritual aspects of care; (3) establishing goals of care, identifying a surrogate decision-maker, and completing advance care planning documentation; and (4) providing care coordination support.

### The Intervention

It is important to note that the nurses delivering the intervention were registered nurses, and the care they provided was within their scope of practice. The nurses assessed the participants using several tools (Multimedia Appendix 1 [6-16]).

The intervention comprised 12 virtual encounters (1 per month) with the participants over a 1-year period, including an initial encounter (session 1) followed by 11 monthly encounters (sessions 2-12).

In session 1, a complete assessment of the participants’ early primary palliative care needs using a set of measures (Table 1) and an assessment of participants’ preferences for care were completed. During this session, the nurses introduced advance care planning and the concept of goals of care. The nurses worked with the participants (and their caregivers if present) to create a list of all the issues they needed help with.

**Table 1.** Protocol used by the nurses to deliver the early primary palliative care intervention over a 12-month period.

	Session type and duration	Agenda and measures
Session 1	Secure initial video encounter for up to 2 hours	Conduct intervention visit and assess the participant using the following measures: Distress Thermometer [1], Katz Activities of Daily Living scale [2], spiritual assessment exploratory questions, social assessment exploratory questions, pain assessment, and depression assessment (Geriatric Depression Scale) Introduction, advance care planning, and discussion on goals of care: life goal list [7], What Matters Most letter advance directive [6], and POLST^a^ [5]
Sessions 2-11	Secure monthly video or audio encounter (based on participant preference) for up to 1 hour	Follow up on supportive care needs (if any in the previous months), administer the Distress Thermometer and pain assessment, and assess for changes in health condition since the last visit Follow up on goals of care and advance care planning Schedule follow-up visit for the following month
Study exit (conducted at session 12)	Secure video or audio encounter (based on participant preference)	Review and summarize the intervention encounters Provide the study team’s contact information for any follow-up

a POLST: Physician Orders for Life-Sustaining Treatment.

After session 1, the nurse presented the participant information and preferences to the study palliative care physician and worked to craft a personalized care plan based on the participants’ needs and expressed wishes.

In sessions 2 to 11, the nurse reviewed the participants’ situation over the previous month and identified any concerns. They completed the Distress Thermometer and pain assessment, used these to explore any underlying issues to be palliated, and discussed those with the study palliative care physician. The study nurses worked with the PCP of each participant to implement the early primary palliative care plan. For example, if the participant reported pain and needed any medications, the study nurse would first consult with the study palliative care physician and then work with the participant to craft a personalized care plan. If the participant and their care partner wanted to contact their PCP, they could do so themselves. If the participant or caregiver wanted the study nurse to contact their PCP on their behalf, the nurse did so. All medical therapies could be prescribed by the individual participants’ PCPs. The nurse continued the discussion about goals of care and advance care planning and helped complete the documentation. If the participant was interested, the nurse discussed the Physician Orders for Life-Sustaining Treatment (POLST) form choices with the participant and helped them identify their preferences. The participant was encouraged to discuss their goals of care with their PCP and finalize their choices. Once the participant and their PCP signed the POLST form, it was uploaded into the electronic medical record, and the original was sent to the participant to post on their refrigerator.

At the end of each encounter, the nurse worked with the participants to identify any issues that needed attention and make plans for follow-up in the next few days. The nurse also scheduled the date for the following month’s session.

On the last follow-up visit (session 12), the nurse reviewed the intervention encounters over the previous year and summarized the issues identified (if any). They provided the study team’s contact information to the participants in the event that they needed further assistance and thanked them for taking part in the study.

### Participants and Recruitment

Potential eligible participants were identified by working with the neurology team. The study coordinators contacted the participants via phone to schedule a screening call to determine eligibility. If eligible, and if the participants and caregivers consented to take part in the study, we scheduled an appointment with the IT expert to help the participants access the video portal through their computer or smartphone. The blinded study evaluators consented all participants and completed all evaluation measures virtually. A total of 200 enrollees who met the inclusion criteria were randomly assigned to the intervention (UC + virtual, nurse-led EPC) or to UC. Consenting caregivers were also allowed to take part as observers along with the participants.

### Inclusion Criteria

Participants were eligible to take part if they were older adults with chronic illnesses living in the community and they had undergone a full evaluation by a multidisciplinary neurology team of experts. The Stanford Alzheimer’s Disease Research Center team assessed the participants’ cognitive status and determined their Clinical Dementia Rating (CDR) scores through consensus. Participants with a composite CDR score of 0 (no symptoms), 0.5 (very mild), 1.0 (mild), or 2.0 (moderate) were eligible to take part in the study.

### Exclusion Criteria

Participants were excluded from the study if they had severe dementia (CDR=3.0) or if they lived in an institutional setting.

### Randomization

After obtaining informed consent, participants were randomized to either the intervention (UC + virtual, nurse-led EPC) or to UC. The study nurses worked with participants randomized to the EPC intervention or their proxies to schedule session 1 of the intervention as a secure virtual video encounter. Sessions 2 to 12 were scheduled monthly video or phone encounters based on participant preference. To the extent possible, the same nurse was assigned to each participant across all sessions to ensure continuity. Evaluators, blinded to the treatment condition, collected the outcome measures from participants in both arms at baseline (at the time of study enrollment) and months 4 and 12. Long-term observational follow-up is currently ongoing for all participants through the electronic health records to track their health care resource use and determine whether the participants received preference-sensitive care at the end of life.

### Primary Outcome Measures

The first primary outcome measure is the number of participants who report supportive care needs in both arms to the blinded evaluators (time points: day 0 and months 4 and 12).

The second outcome measure is completion of conversations on goals of care and documentation of advance directives and the POLST [8] in the electronic health records (time points: daily during the intervention period).

The third outcome measure is end-of-life resource use (hospital admissions and days and emergency visits). We hypothesize that this will be lower for the participants in the intervention group and that, compared to those in the control group, the participants in the intervention group are more likely to receive goal-concordant care at the end of life. In both arms, blinded evaluators will track the participants’ end-of-life outcomes by monitoring their electronic health records and through interviews with their caregivers after the death of the participants (time points: at 1 year and then annually).

### Secondary Outcome Measures

#### Changes in Edmonton Symptom Assessment Scale Score

The Edmonton Symptom Assessment Scale [9] was designed to assist in the assessment of 9 symptoms common in patients: pain, tiredness, nausea, depression, anxiety, drowsiness, appetite, well-being, and shortness of breath. The severity at the time of assessment of each symptom is rated on a numerical scale from 0 to 10, with 0 meaning that the symptom was absent and 10 meaning that it was of the worst possible severity. The investigators hypothesize that participants with cognitive impairment will have higher scores than those with normal cognition (time points: day 0 and months 4 and 12)*.*

#### Changes in Patient Activation Measure Over Time

The Patient Activation Measure (PAM) [10] is a 3-item survey that assesses a person’s underlying knowledge, skills, and confidence integral to managing their own health and health care. The PAM segments individuals into 1 of 4 activation levels along an empirically derived 100-point scale. Each level provides insight into health-related characteristics, including attitudes, motivators, and behaviors. Individuals in the lowest activation level do not yet understand the importance of their role in managing their own health and have significant knowledge gaps and limited self-management skills. Individuals in the highest activation level are proactive with their health, have developed strong self-management skills, and are resilient in times of stress or change (time points: day 0 and months 4 and 12)*.*

#### Quality of Life in Alzheimer’s Disease

The Quality of Life in Alzheimer’s Disease (QOL-AD) scale [11] is a valid and reliable scale that is brief (13 items) and has good content validity, criterion concurrent validity, and construct validity. Interrater reliability was strong, with all Cohen κ values of >0.70. Internal consistency was good, with a Cronbach α of 0.82. Some people with severe dementia and a Mini-Mental State Examination score as low as 3 have been able to satisfactorily complete the QOL-AD (time points: day 0 and months 4 and 12)*.*

### Statistical Analysis

To compare the EPC intervention and UC groups, for ordinal outcome measures, we will use a hierarchical linear model that assumes that the outcome *O* for each participant *p* at a time *t* is a linear function with an intercept *i* and slope *s*: *O*(*p*,*t*) = *i*(*p*) + *s*(*p*)ln(*t*+1) + e(*t*,*p*), where *t* is the time of measurement (measured from baseline) and *e* is an error term. The intercept for each participant (*i*[*p*]) is the true baseline response, with randomization having the same distribution in both groups. The slopes of the groups reflect the impact of the intervention or of UC over the course of treatment. Both intercepts and slopes are assumed to have a bivariate distribution in the population of participants, and the error terms within participants are assumed to be intercorrelated according to an autoregressive covariance function (outcomes closer together are assumed to be more highly correlated than those more distal to each other). In addition, ln(*t*+1) is used rather than *t* because outcomes in randomized clinical trials are more likely to have a “fishhook” relationship to time *t*, with more rapid changes early on than later.

For the power calculation, the null hypothesis to be tested is that the slopes in the EPC group have the same distribution as those in the UC group (ie, that there is no effect of EPC relative to UC). The significance level is 5%, with no adjustment for multiple testing because the outcomes chosen tend to be independent of each other. For 80% power to detect a moderate effect size with a 2-tailed 5% test, the sample size per group is approximately 63. However, we recruited a total of 100 participants per arm (200 in total) as we anticipated that the number of participants with a CDR score of 0 and at risk of cognitive impairment and those with a CDR of 0.5, 1, or 2 would be similar, allowing for within-group analysis.

### Ethical Considerations

#### Ethics Approval and Informed Consent

This study was approved by the Administrative Panel on Human Subjects in Medical Research at Stanford University. Informed consent was obtained by the study evaluators at the start of participation.

#### Planned Procedures for Protection of Confidentiality

All data will be coded using participant number to ensure anonymity of participation. Data will be kept in locked cabinets and secured computer files. Online data will be hosted on a secure server at Stanford University and available through a double authorization protocol for maximal security. Research records will be kept confidential to the level allowed by law. The findings of this research may be published; however, neither the name nor identity of the participants or their caregivers will be used in any publications. Only the study manager and database manager had access to the research database.

#### Compensation Details

All participants in both arms will receive a US $25 stipend after they complete the evaluation measures at each time point (baseline and months 4 and 12).

#### Potential Risks of the Proposed Study to the Participants

Primary palliative care consultation is known to be beneficial and not risky. We highlight that all the medical treatments will be conducted by the individual participants’ PCPs, ensuring careful care coordination. The participants’ PCPs served as the attending physicians and signed the POLST form based on the participants’ goals of care. As the participants’ care partners (if available) were involved in the early primary palliative care plan, and as the individual participants’ PCPs provided all medical therapies, there was minimal risk to the participants.

#### Potential Benefits of the Proposed Study to the Participants and Others

It is expected that the risk-to-benefit ratio of this project will be favorable. At worst, participants could drop out and receive UC. All study participants were provided with a courtesy stipend for taking part in the study. Beyond the benefit for the individual participants, this study has a transformational potential as it could identify the common early primary palliative care needs of participants with mild cognitive impairment and mild and moderate dementia living in the community. If successful, this is a scalable, low-risk virtual intervention that is participant-centered and family-oriented, with minimal risks to participants.

#### Monitoring Procedures and Treatment for Adverse Reactions

Any adverse reactions must be reported to the Stanford Institutional Review Board as per our standard protocol. At any point in the protocol, a treatment failure may be declared, and the participants can opt out of the intervention at any time. Participants were free to withdraw their consent and discontinue participation at any time without prejudice to them or effect on their (the participants’) medical care.

## Results

This trial was initiated in December 2019. As of October 2025, we have recruited 200 participants, and 100 (50%) have completed the intervention. We are tracking the participants in the study on an ongoing basis. We anticipate that the participants in the intervention arm will receive goal-concordant care at the end of life, and their health care resource use will be lower than that of the participants in the UC arm.

## Discussion

### Expected Findings

There is a growing shortage of palliative care workers. There is also an escalating need for early implementation of primary palliative care for older adults in the community with dementia and other serious illnesses. The registered nurse workforce can be trained using the ELICIT intervention model to provide early primary palliative care within the scope of nursing.

Our trial is notable because most existing research focuses on the palliative needs of patients who have severe dementia or are in an institutional setting [17-21]. To our knowledge, this is the first nurse-led, virtual intervention that provides early primary palliative care for older adults with cognitive impairment living in the community. Registered nurses provide the bulk of care for patients enrolled in hospice. However, currently, registered nurses do not provide early primary palliative care to patients. While a registered nurse cannot function as an independent provider, trained nurses are well able to provide early primary palliative care in collaboration with a patient’s PCP. Once we identify the scope of practice for registered nurses in providing early primary palliative care, nurses can be trained using our intervention. Training the nurse workforce to provide early primary palliative care will greatly expand access to these services and enable patients to receive primary palliative care early in their illness trajectory.

Older adults are often excluded [22] from randomized clinical trials. This randomized controlled trial has recruited 200 community-dwelling older adults with chronic illnesses and varying levels of cognitive impairment, and currently, we are following the participants on an ongoing basis.

There is limited research and understanding regarding the primary palliative care needs of patients in the early stages of cognitive impairment, making it difficult to provide timely and effective care interventions. Experts have noted that primary palliative care is a fundamental aspect of high-quality care for patients with dementia [23]. While primary palliative care is appropriate for persons living with dementia at any stage, challenges exist in identifying and assessing their unique physical, psychological, social, and spiritual needs, particularly due to the delayed diagnoses, uncertain prognosis, and difficulties in communication and decision-making. In this trial, we tracked the supportive care needs expressed by all the participants to the blinded evaluators. On completion of this study, we will have a better understanding of the primary palliative care needs of patients with mild and moderate cognitive impairment who are living in the community.

Older adults with moderate and severe dementia often lose their ability to participate in discussions on goals of care and shared decision-making. Eliciting the patients’ values and goals of care and obtaining anticipatory guidance for their future care is an integral part of high-quality care [24]. Studies have shown that patients tend to choose less aggressive interventions when they have an opportunity to participate in shared decision-making and document their preferences [25-27]. It is also known that patient-designated and next-of-kin surrogates incorrectly predict patients’ end-of-life treatment preferences [28]. There is a window of opportunity early in the trajectory of cognitive decline when the patient retains mental clarity and decisional capacity. Discussions on goals of care should be conducted during this window while patients are still able to voice their values and preferences for care [29]. While the patients’ goals and preferences may change over time, documenting goals of care and completing advance care planning will serve to establish a baseline of their preferences while they still have the ability to express them.

### Study Limitations

This study had the following limitations. First, the COVID-19 pandemic started at the exact time when we enrolled our very first participant, and we rapidly redesigned our trial into a virtual intervention. If COVID-19 had struck even a few months later, we would have been in the middle of our trial and forced to stop the study as our original intervention and evaluation were supposed to be in person. In preparation for the virtual study, we piloted all the study measures with a few participants before the start of enrollment. Our pilot participants reported challenges with completing the Edmonton Symptom Assessment Scale, PAM, and QOL-AD measures virtually as some questions had reverse wording and all had Likert-scale response options that made it hard for them to answer. As the measures were part of our original proposed study, we retained them as secondary measures. Second, in this study, early primary palliative care was provided by registered nurses. Trained registered nurses can assess participants and identify their medical and psychosocial needs, provide care coordination and education, and conduct conversations on goals of care; however, they cannot independently make a diagnosis or prescribe medications. While the nurses were able to fulfill the psychoeducation and care coordination needs of the participants, they had to work with the participants’ PCPs to fulfill all their medical needs. This can be seen as a study limitation, but we believe that this is also a strength as defining the scope of registered nurses as encompassing the provision of primary palliative care will inform the path forward as we work to expand the primary palliative care workforce. If effective, this intervention might be an innovative solution to offset the growing shortage of palliative care workers. Nurses trained in this intervention may be able to become the backbone of the primary palliative care workforce in the future. Third, this study was a virtual, ELICIT instituted early in the trajectory of cognitive impairment, and its long-term impact is currently unknown. We are conducting long-term observational follow-up of study participants to better understand whether the intervention has any impact on the end-of-life care of the study participants and whether it resulted in goal-concordant care.

### Importance of Knowledge to Be Gained

Currently, very little is known about the early primary palliative care needs of patients with cognitive impairment living in the community. If successful, this trial will delineate the common early primary palliative care needs of patients at risk of cognitive impairment and those with mild cognitive impairment and mild and moderate dementia. Such knowledge will allow future interventions to better support persons living with cognitive impairment in the community.

## Supplementary material

10.2196/75082 Multimedia Appendix 1 Study protocol and study measures.

## Abbreviations

CDR: Clinical Dementia Rating
ELICIT: early primary palliative care intervention
EPC: early palliative care
PAM: Patient Activation Measure
PCP: primary care provider
POLST: Physician Orders for Life-Sustaining Treatment
QOL-AD: Quality of Life in Alzheimer's Disease
UC: usual care
WMM: What Matters Most letter advance directive
