# Supplementary material for: Virtual, Nurse-Led Early Primary Palliative Care Intervention (ELICIT) for Community-Dwelling Older Adults With Cognitive Impairment: Protocol for a Randomized Controlled Trial
Source: JMIR Res Protoc. 2025 Dec 24;14:e75082. doi: 10.2196/75082 (PMC12731548; doi:10.2196/75082)
Supplement: Multimedia Appendix 1 [file resprot-v14-e75082-s001.docx]

**STUDY TOOLS AND MEASURES:**
**NURSE-LED  PRIMARY PALLIATIVE CARE INTERVENTION:**

Study goal: Our study seeks to better understand the scope and impact of a virtual, nurse-led, primary palliative care intervention on chronically-ill older adults living in the community who have a diagnosis of cognitive impairment or are at risk for it. In our study, patients were randomized to either {usual care} or {usual care+ a virtual, primary palliative care consultation provided by a trained nurse (RN) over a 12-month period} .

Experts have identified that the four domains[1] of primary palliative care are as follows : (a) assessing and palliating physical symptoms, (b) assessing and managing psychological, social, cultural, and spiritual aspects of care, (c) establishing goals of care, identification of a surrogate decision maker and completion of advance care planning documentation; and (d) providing care coordination support. Nurses provided primary palliative care for the persons randomized to the intervention arm. The intervention was designed to ensure that it was within the scope of nursing.

**Intervention procedure:** Trained nurses provided primary palliative care consultation within the practice scope of a registered nurse. The nurses were trained to utilize a standard protocol to do a complete assessment of the patient. At first, the nurses used the distress thermometer to identify and describe the amount of stress the patient has been experiencing, assess for practical problems, family problems, emotional problems, and spiritual/religious concerns. Then they completed a spiritual assessment and a social assessment to better understand the patient support system and community resources available to them. Comprehensive measures were used to carefully catalog the patient’s activities of daily living, pain and non-pain symptoms. Once they had conducted a full assessment as per this protocol, each nurse was able to get a clear understanding of the patient’s primary palliative needs. They worked with the patient and their caregiver to create a list of all the issues they needed help with. The nurse then presented the patient’s case to the study palliative physician and devised a care plan based on the patient’s preferences and needs. For any medical consultation or treatment needs, the nurses worked with the patient and their primary care provider to attend to their supportive care needs. The nurses also helped with care-coordination and patient and family education during the 12-month intervention period for each patient.

Using the Stanford Letter Project life goal list form, the nurses explored the patients desires and goals for the future and help them make a list of the things that they wanted to accomplish in their lifetime. Next, using the Stanford Letter Project What Matters Most tool (see below), the nurses were able to elicit the patient’s goals and preferences for care. Through detailed discussion, the nurses developed a good understanding of the patient’s goals of care. For interested patients, they helped them complete the What-Matters-Most letter advance directive form. They helped the patient review the POLST form and the choices it contained. They then recommended that the patient schedule an appointment with their primary care provider to discuss their care goals and finalize their choices. Once the patient and the doctor signed the POLST form, it was uploaded into the patient’s electronic health records.

Please find below the tools used by our nurses to assess the patient and provide the primary palliative care consultation.

**MEASURES AND TOOLS USED BY THE NURSES:**


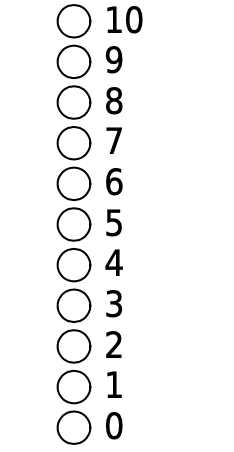
Distress Thermometer and Problems List for Patients [2]

1)Instructions: First please select the number (0-10) on the scale to the right of the image that best

describes how much distress you have been experiencing in the past week including today.

Second, please indicate if any of the following has been a problem for you in the past week including today. Be sure to check YES or NO for each.

**Practical Problems**

2) Childcare O YES O NO

3) Housing O YES O NO

4) Insurance/financial O YES O NO

5) Transportation O YES O NO

6) Work/school O YES O NO

7) Treatment decisions O YES O NO

**Family Problems**

9) Dealing with children O YES O NO

10) Dealing with partner O YES O NO

11) Ability to have children O YES O NO

12) Family health issues O YES O NO

**Emotional Problems**

13) Depression O YES O NO

14) Fears O YES O NO

15) Nervousness O YES O NO

16) Sadness O YES O NO

17) Worry O YES O NO

18) Loss of interest in usual activities O YES O NO

**Spiritual/religious concerns**

19) O YES O NO

**Physical Problems**

20) Appearance O YES O NO

21) Bathing/dressing O YES O NO

22) Breathing O YES O NO

23) Changes in urination O YES O NO

24) Constipation O YES O NO

25) Diarrhea O YES O NO

26) Eating O YES O NO

27) Fatigue O YES O NO

28) Feeling Swollen O YES O NO

29) Fevers O YES O NO

30) Getting around O YES O NO

31) Indigestion O YES O NO

32) Memory/concentration O YES O NO

33) Mouth sores O YES O NO

34) Nausea O YES O NO

35) Nose dry/congested O YES O NO

36) Pain O YES O NO

37) Sexual O YES O NO

38) Skin dry/itchy O YES O NO

39) Sleep O YES O NO

40) Substance abuse O YES O NO

41) Tingling in hands/feet O YES O NO

**42) Other Problems:**

______________________________________________________________________________________________________

**
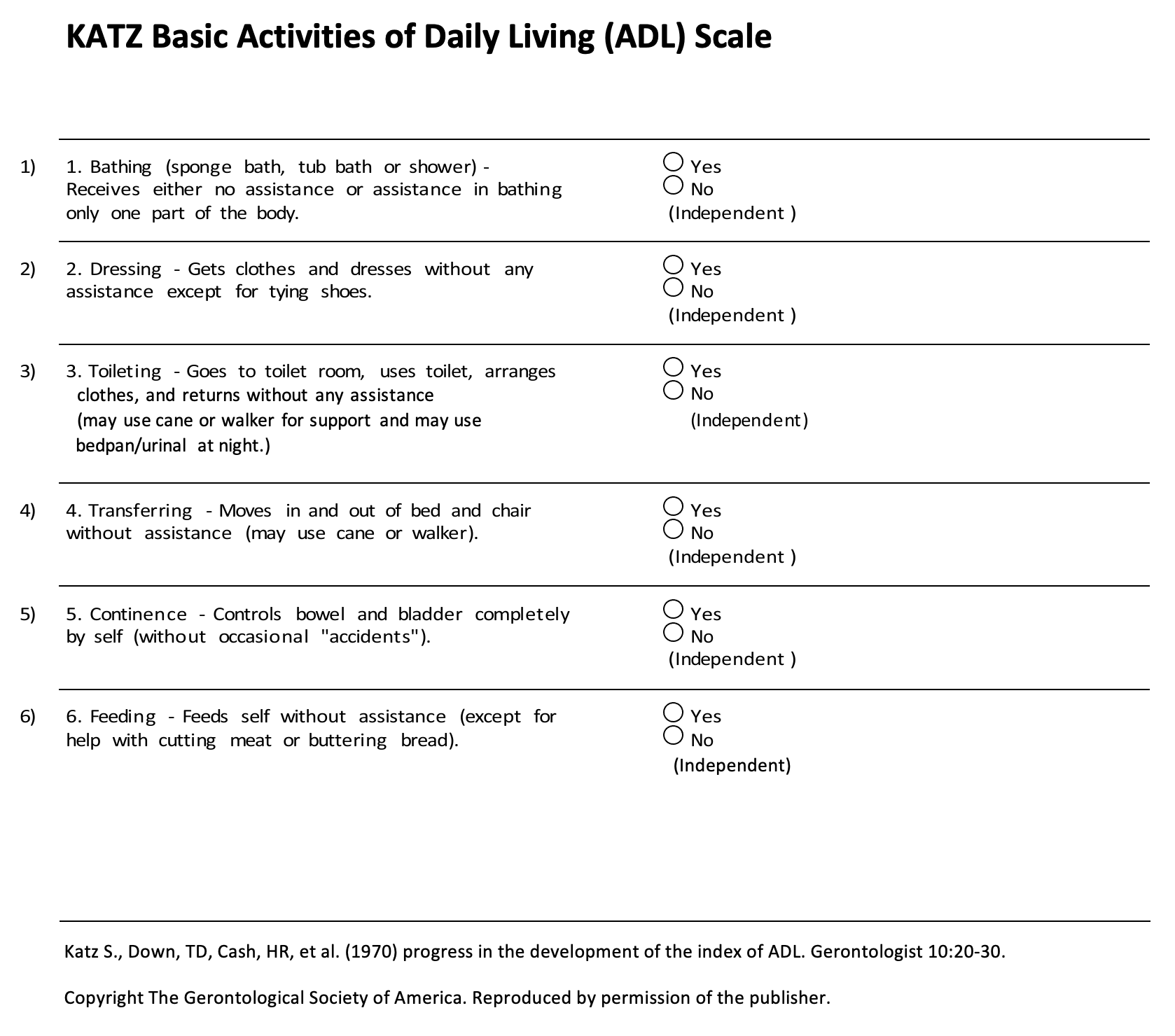
KATZ Basic Activities of Daily Living (ADL) Scale[3]**


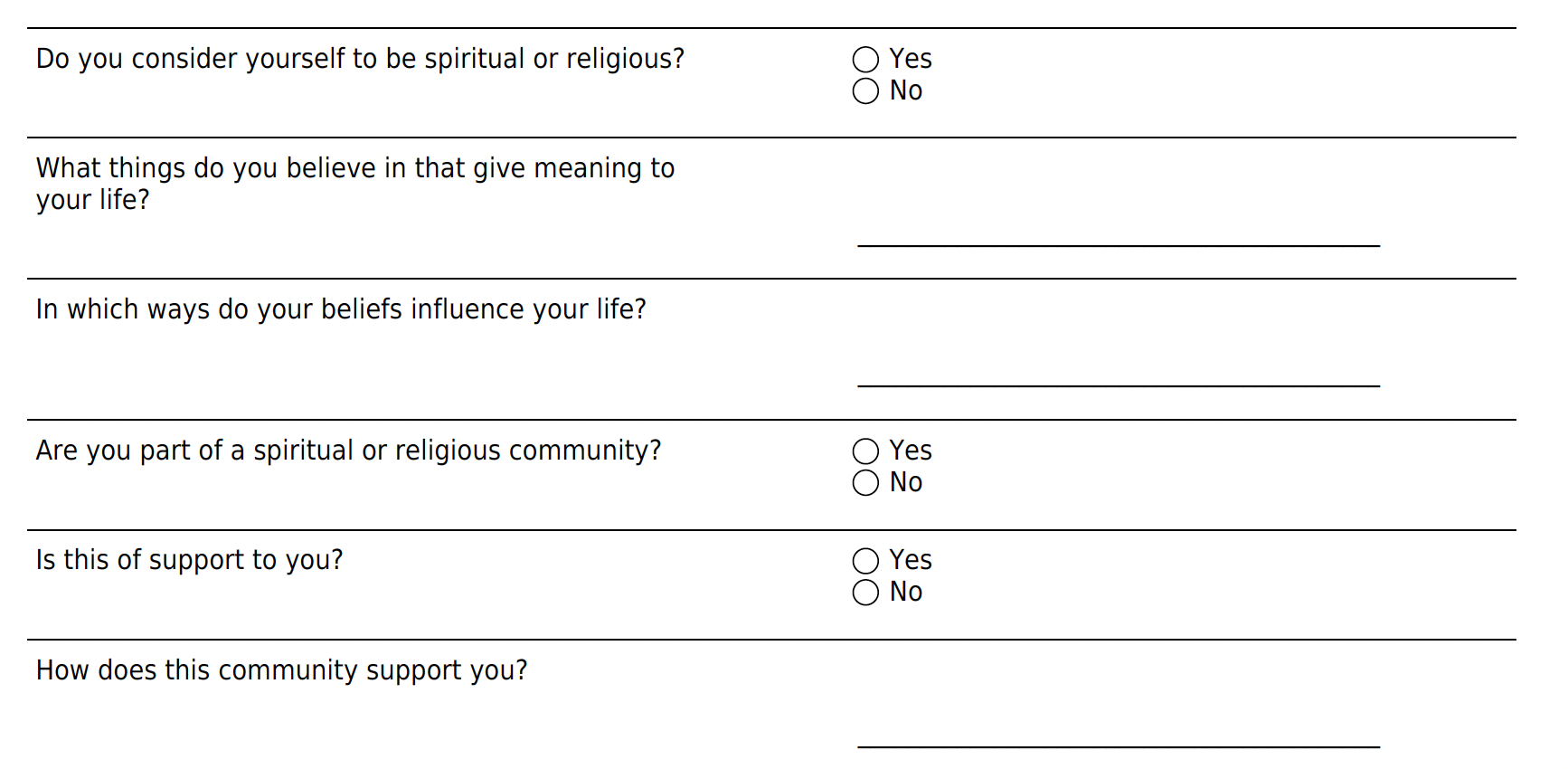
**Spiritual Assessment Exploratory Questions**


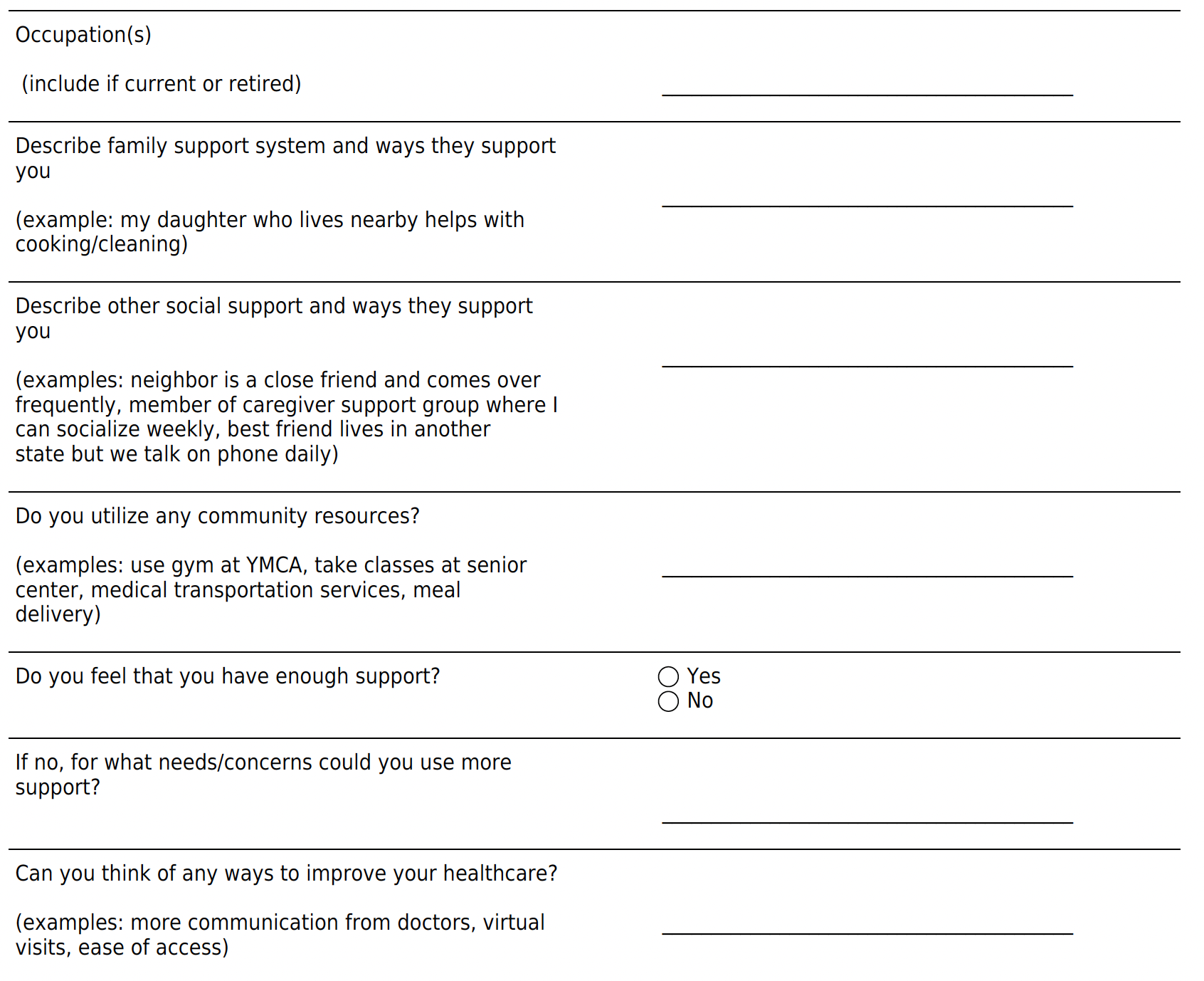
**Social Assessment Exploratory Questions**

**Pain Assessment**


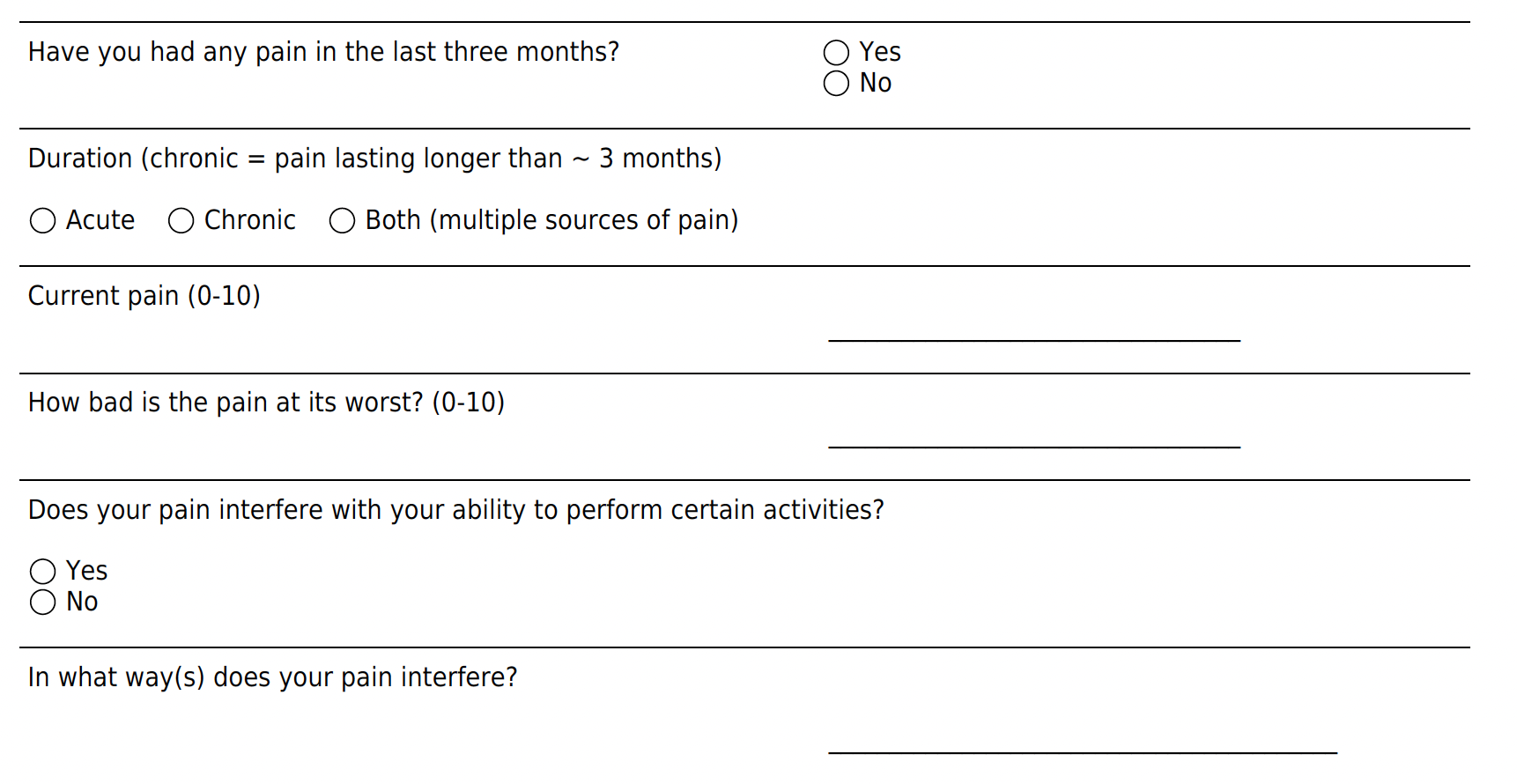

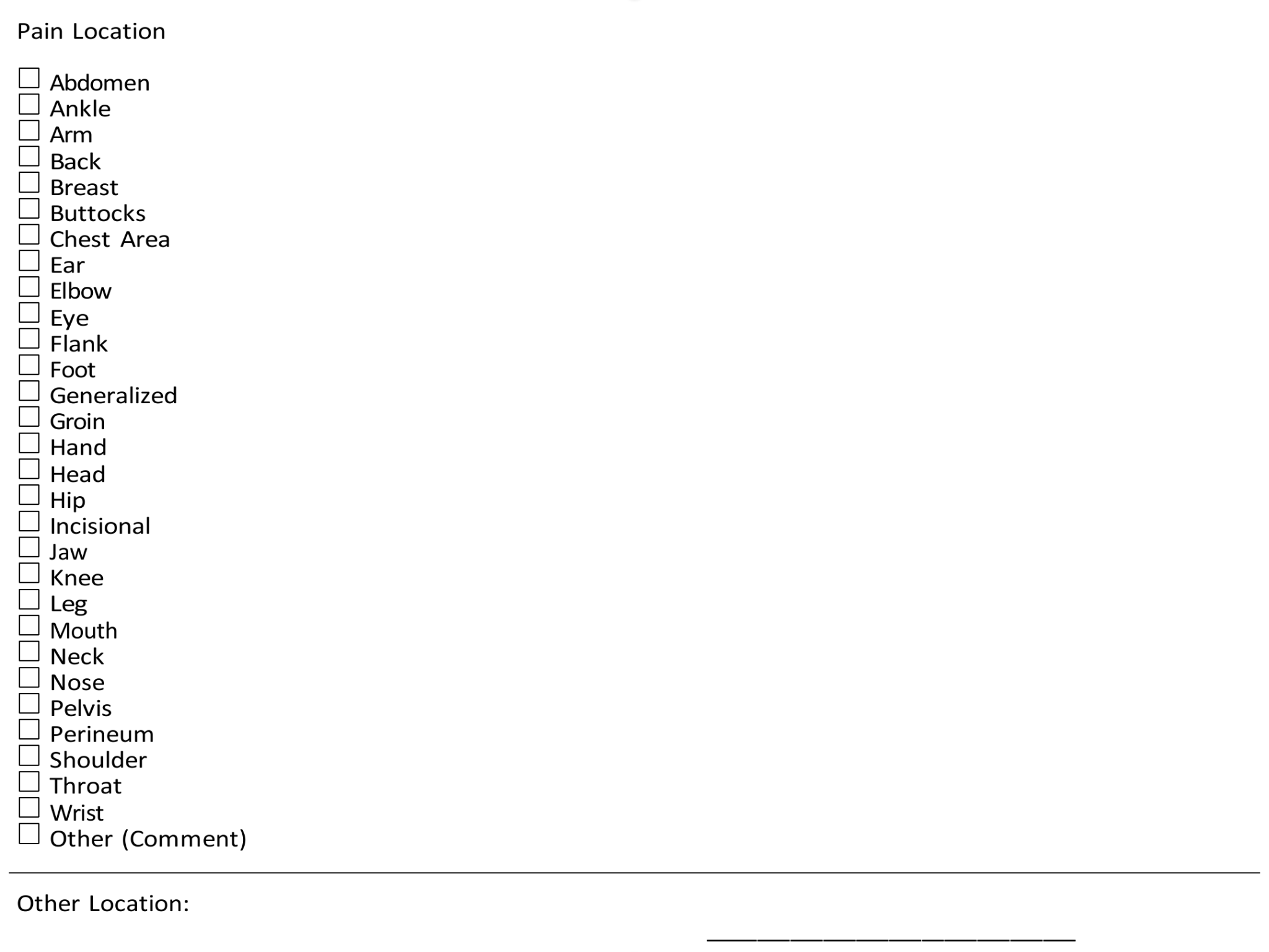


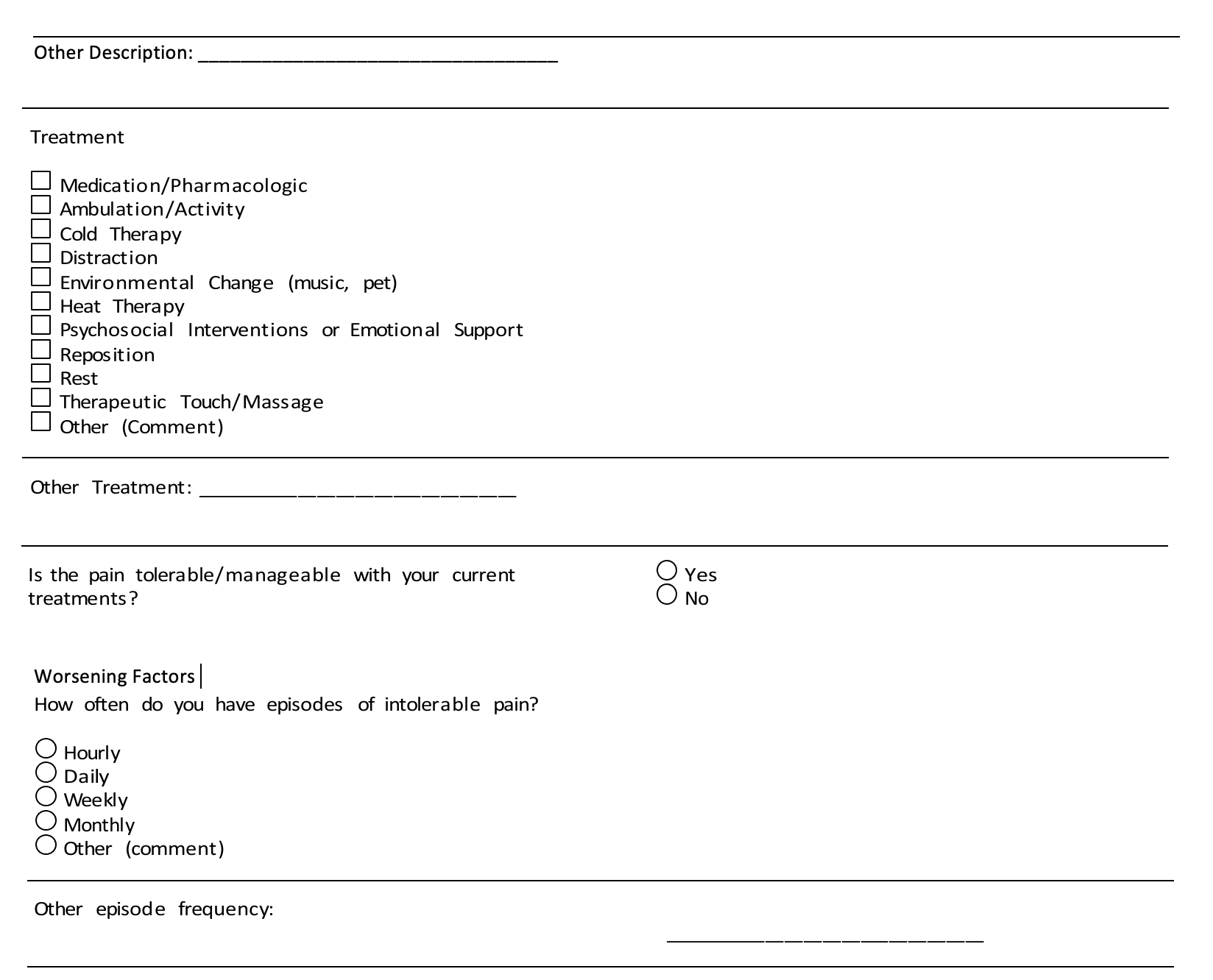

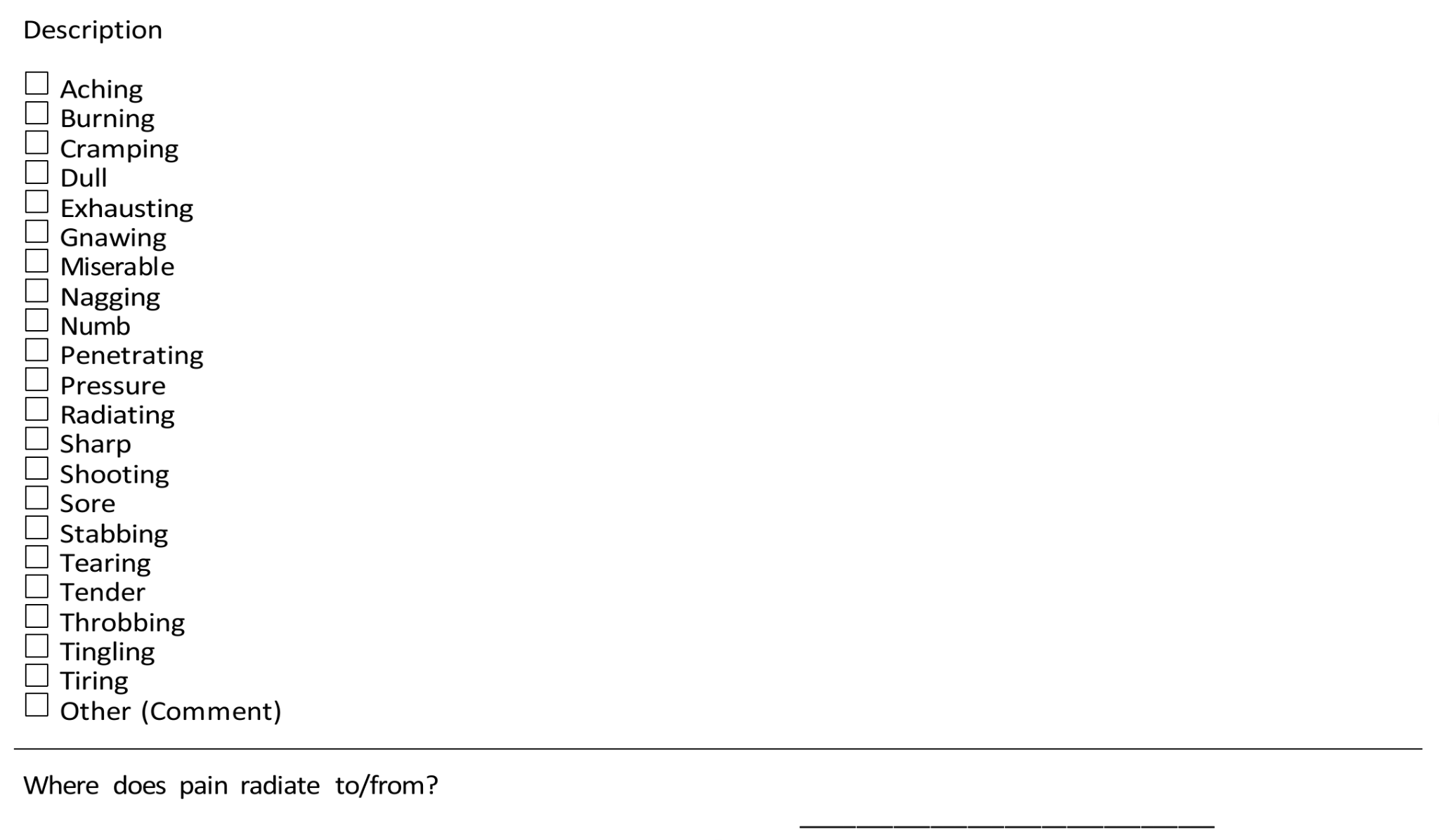


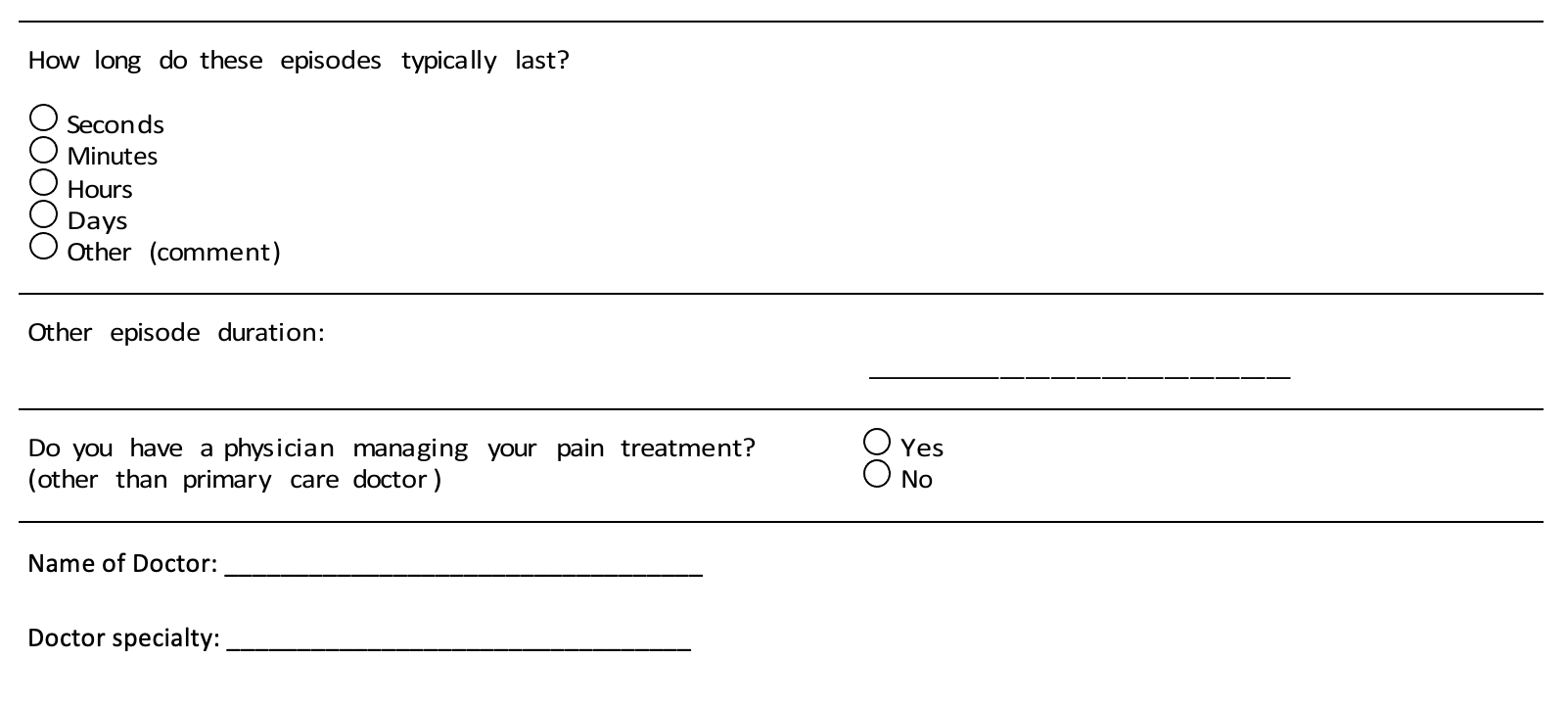


**Depression assessment using the Geriatric Depression Scale [4]**


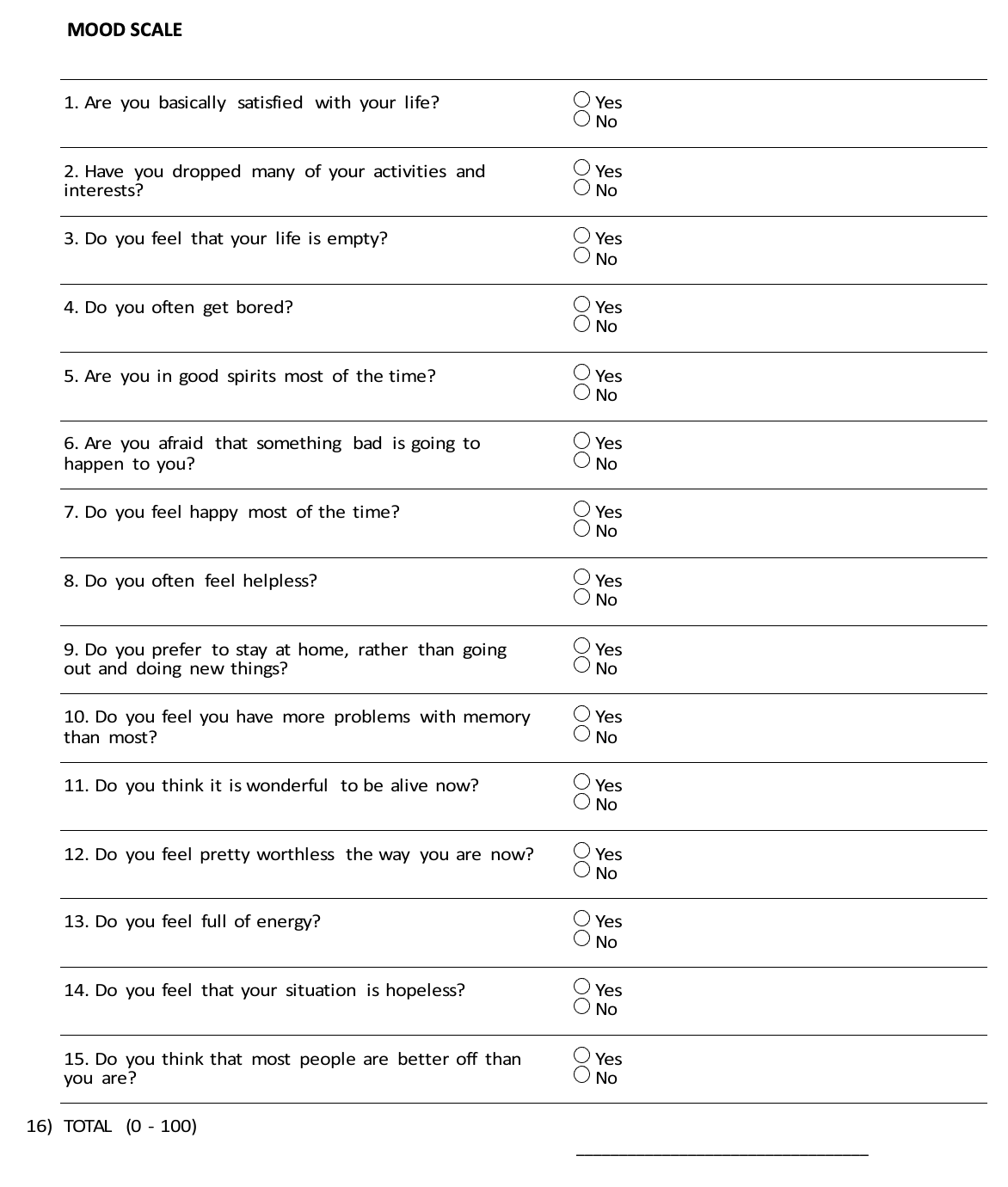


**Life Goal List[5]**

^
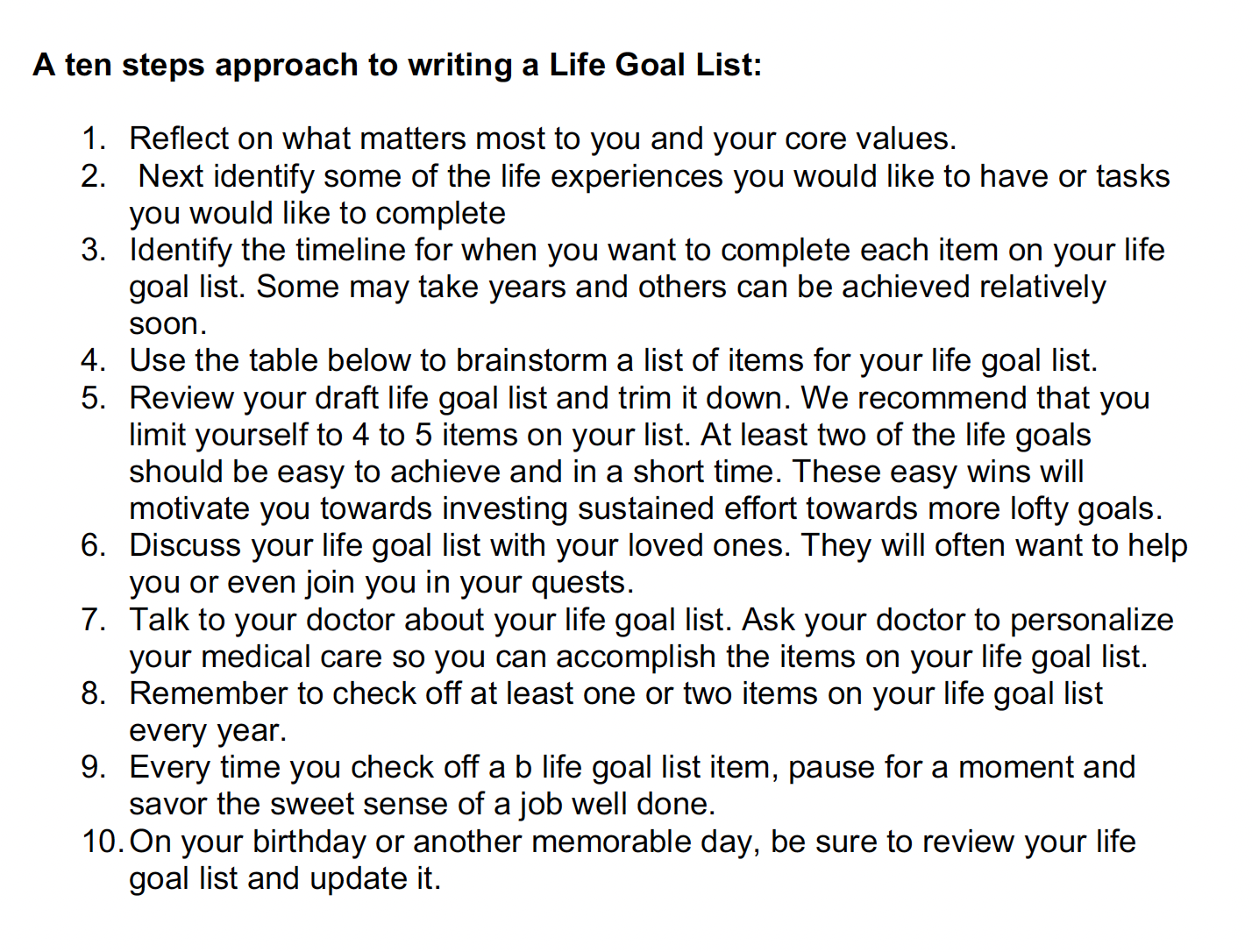
^

^
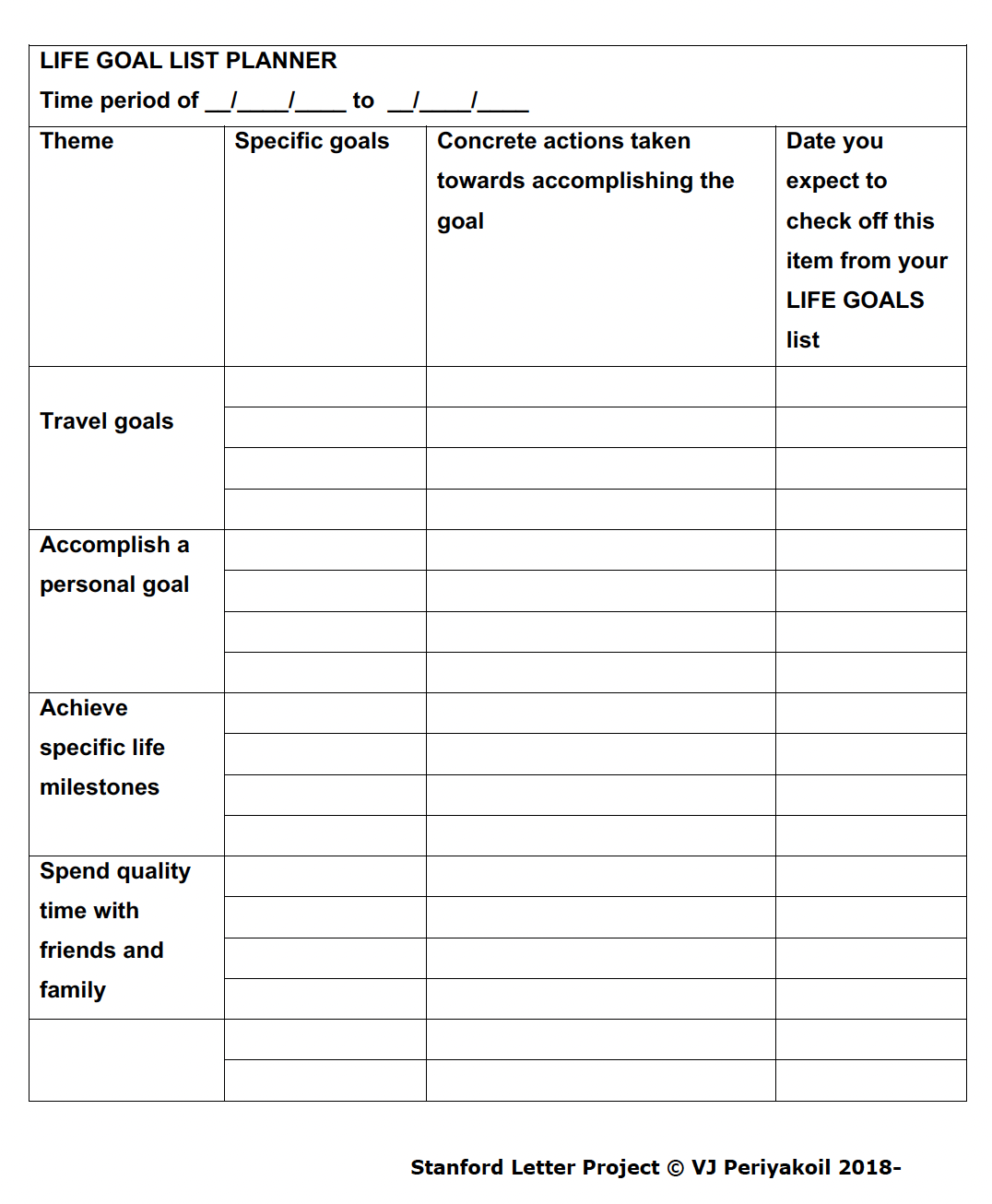
^

^
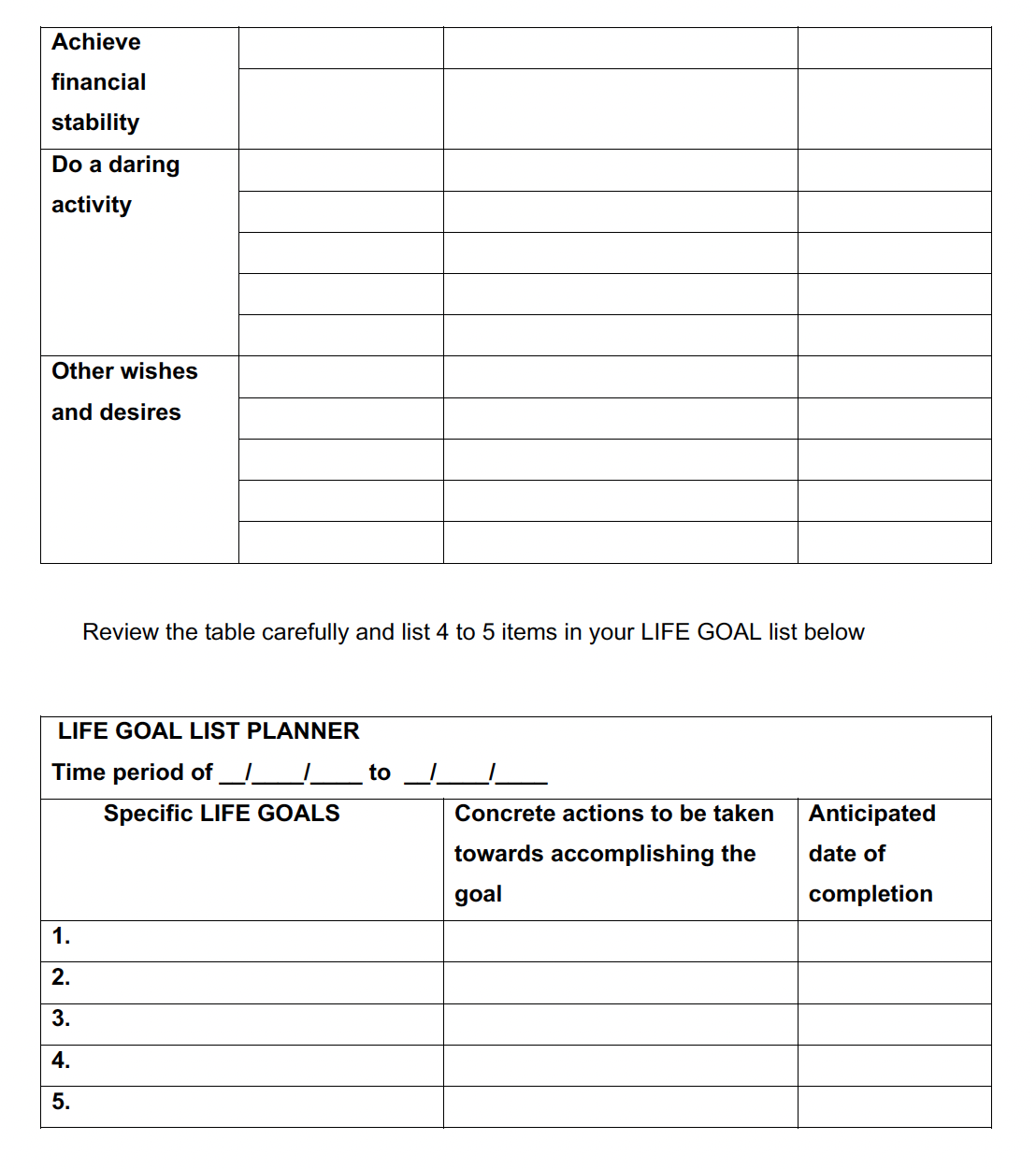
^

What Matters Most Letter Advance Directive[6]


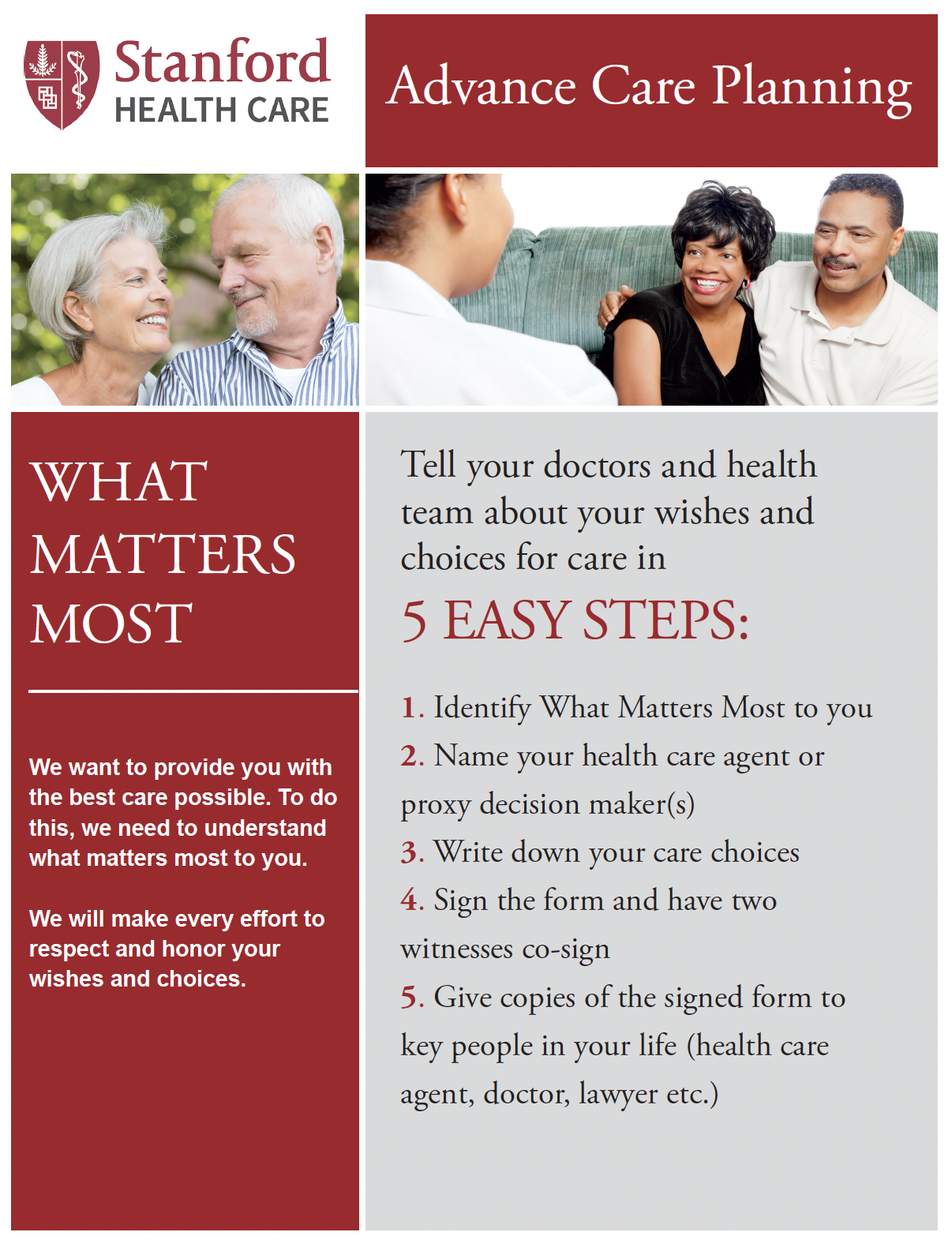


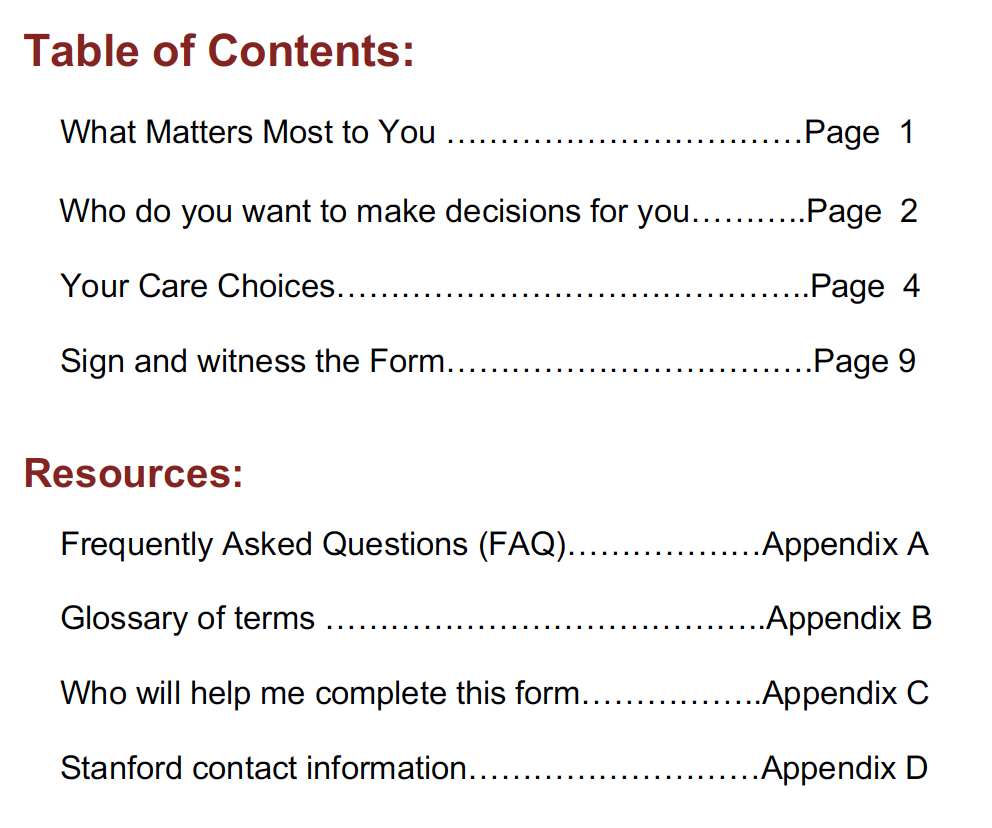


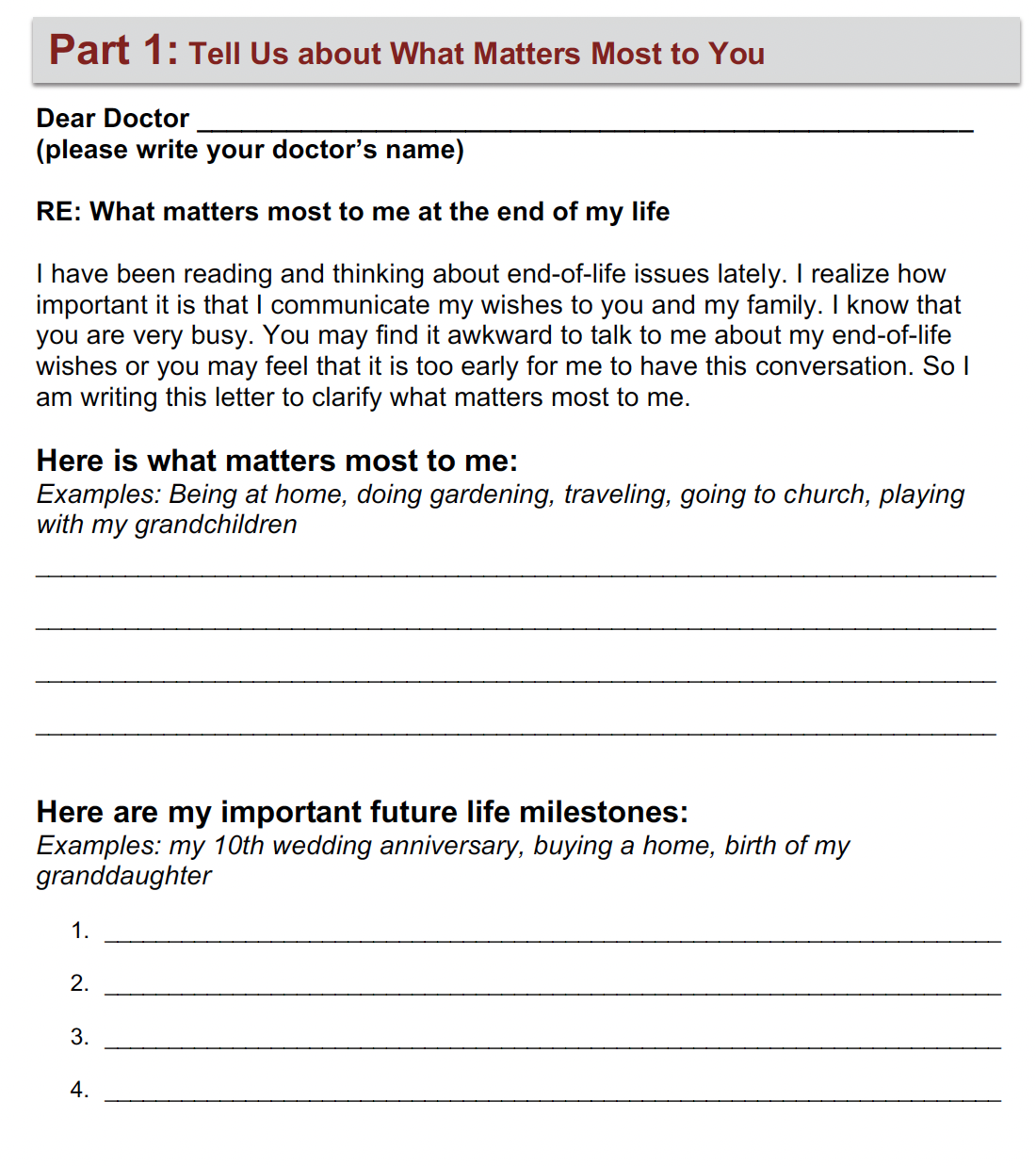


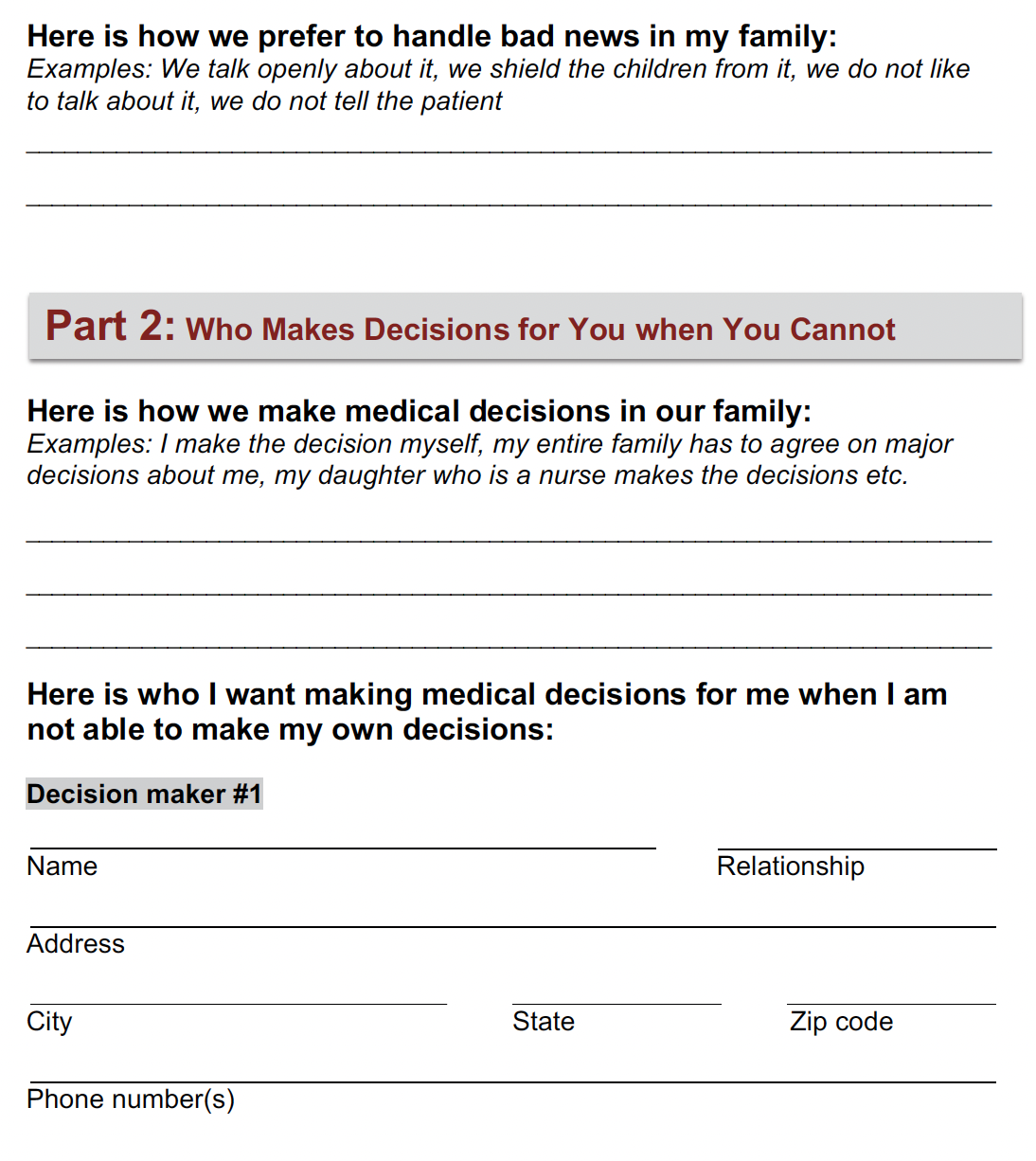


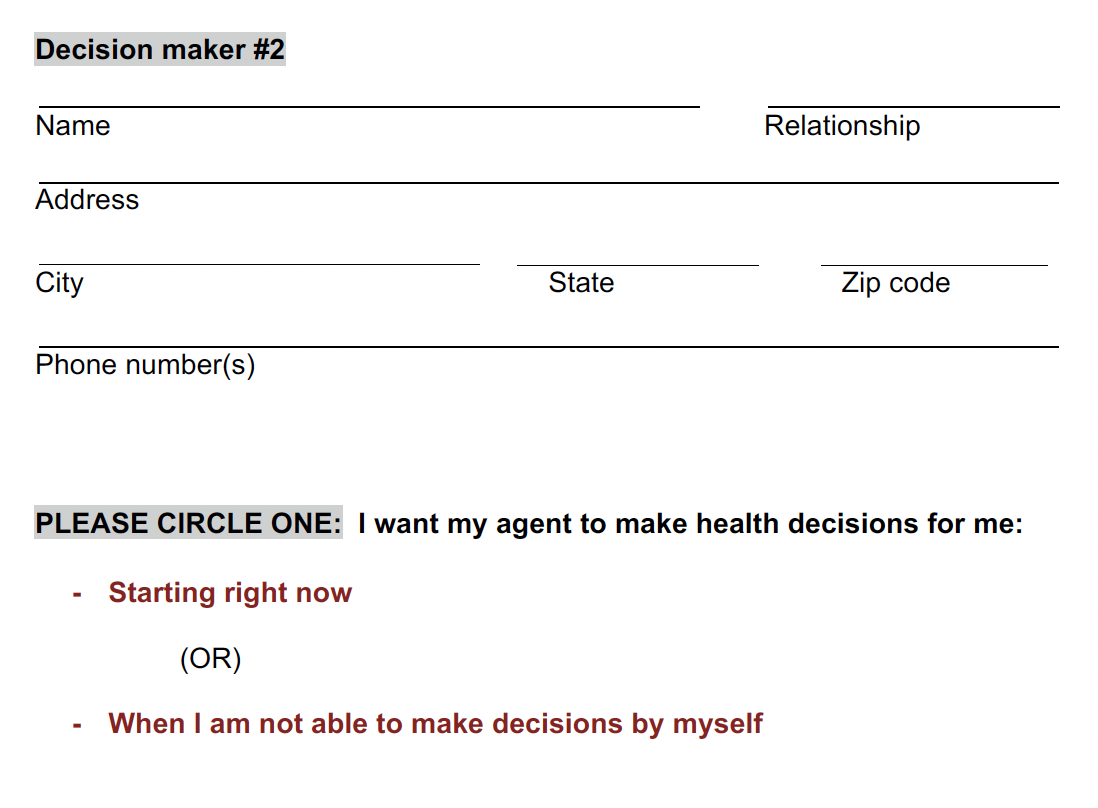


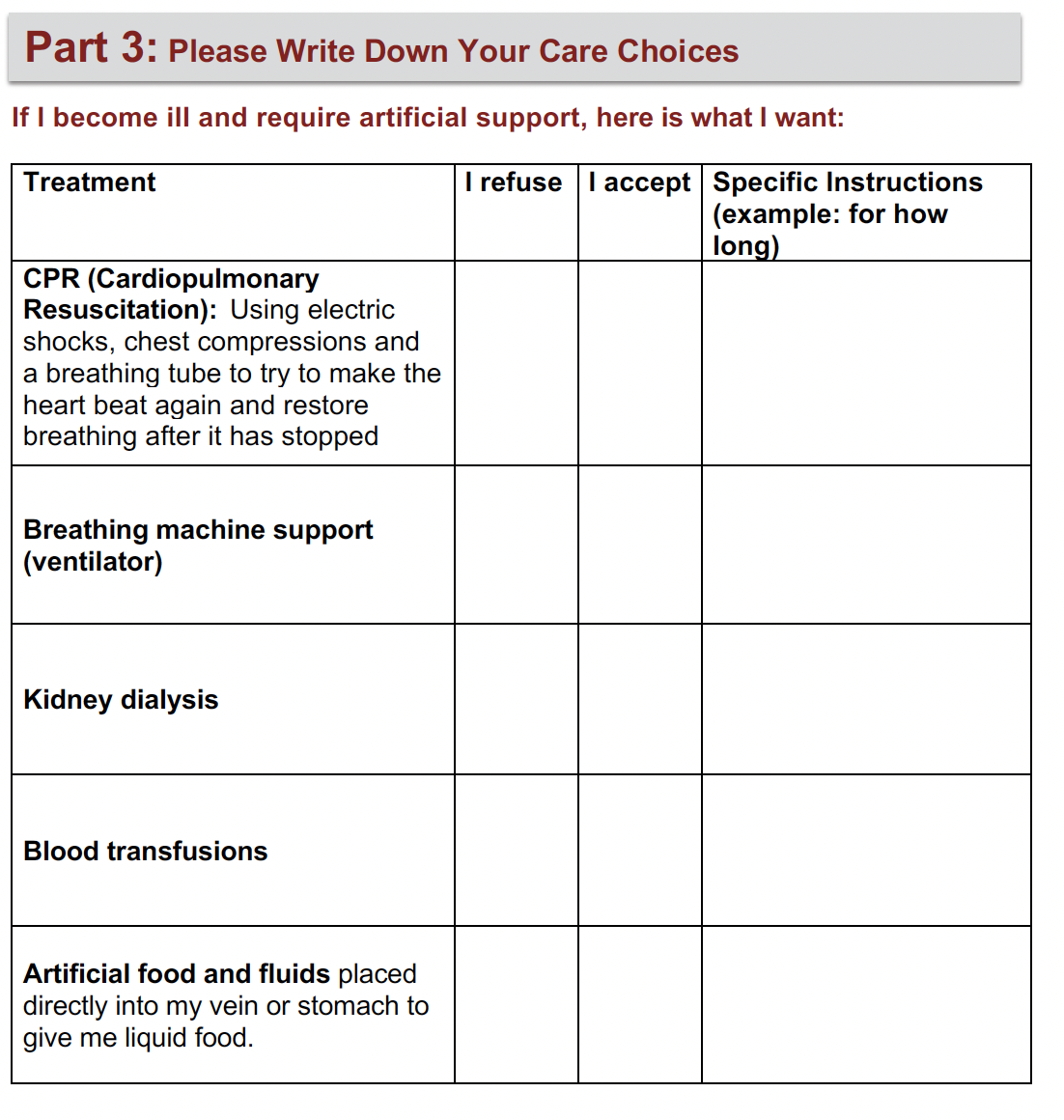


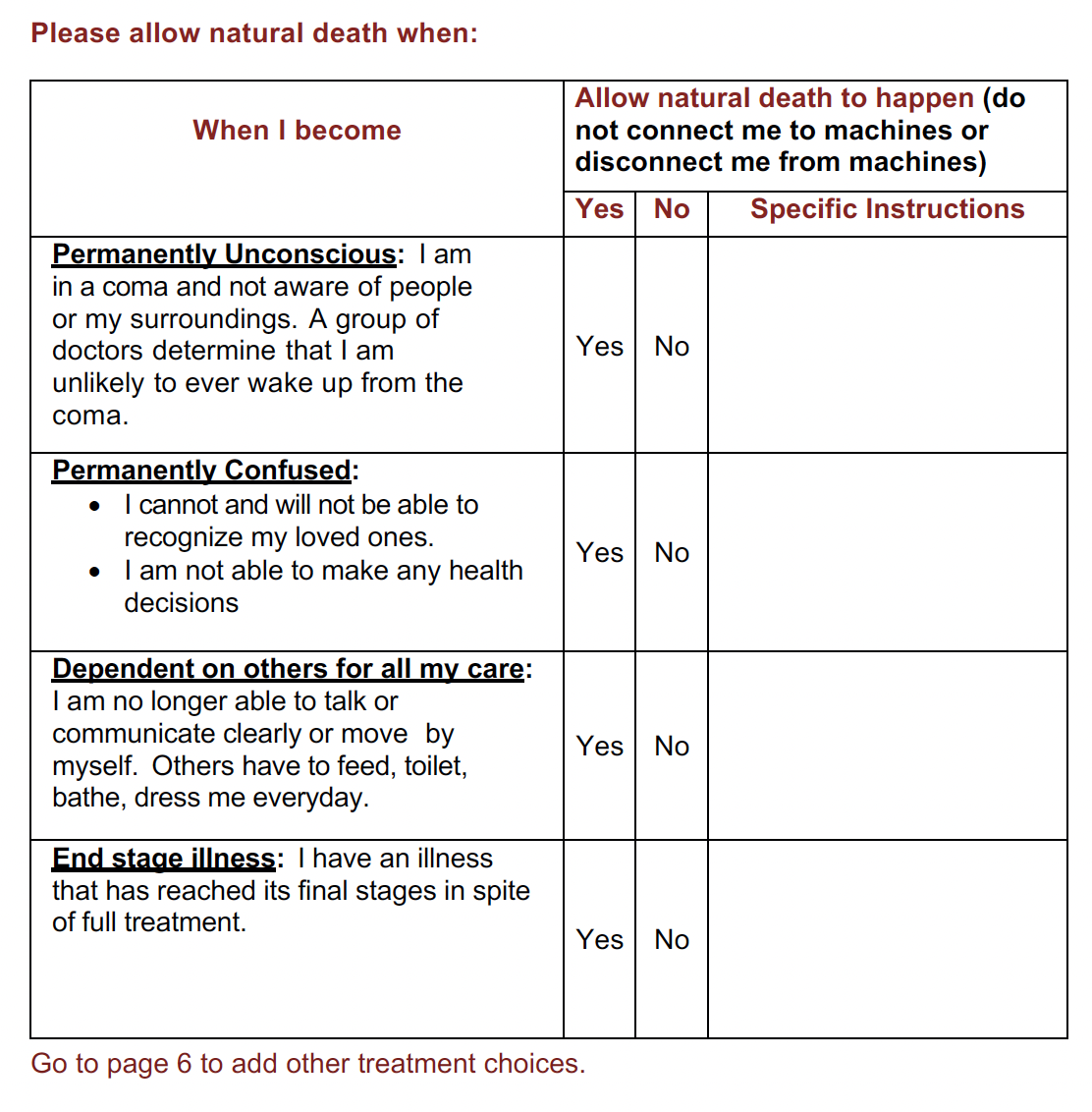


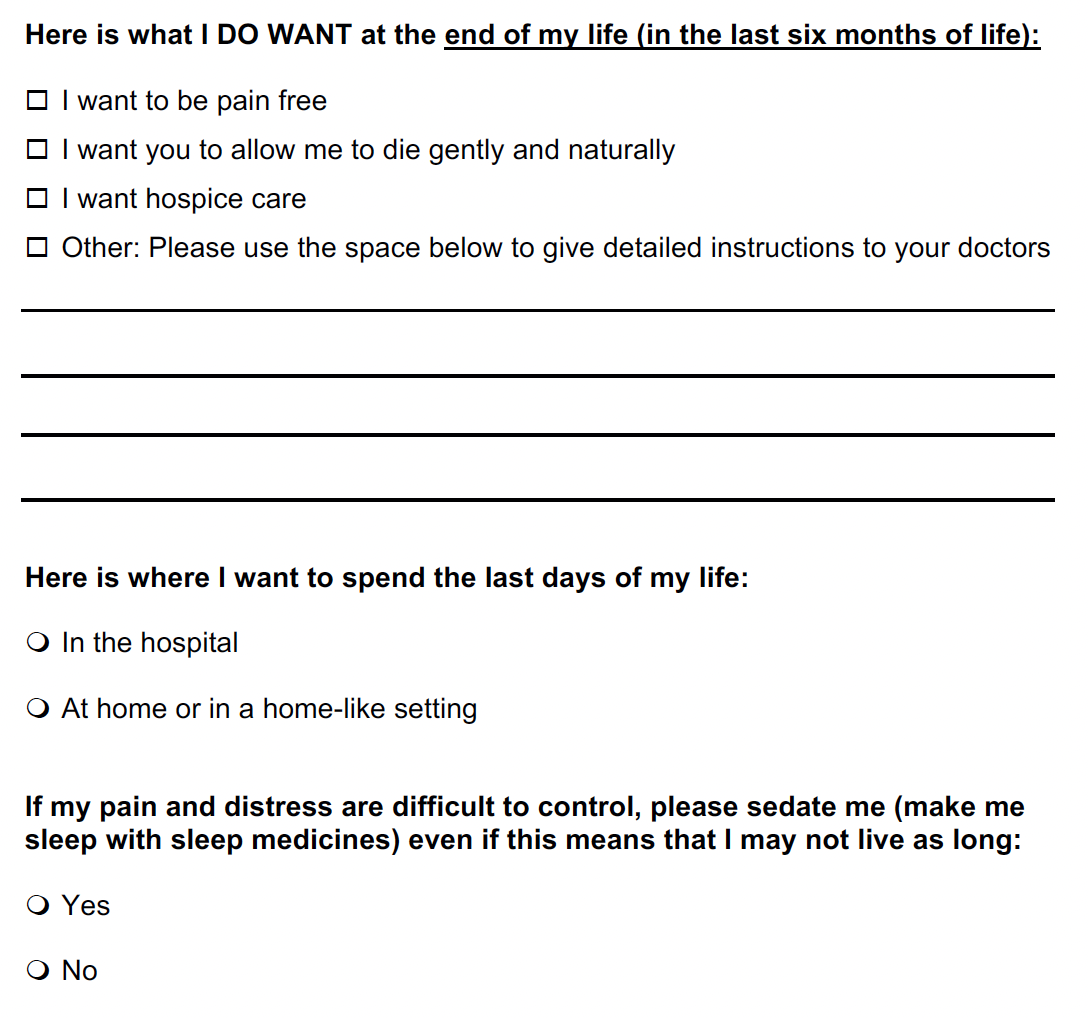


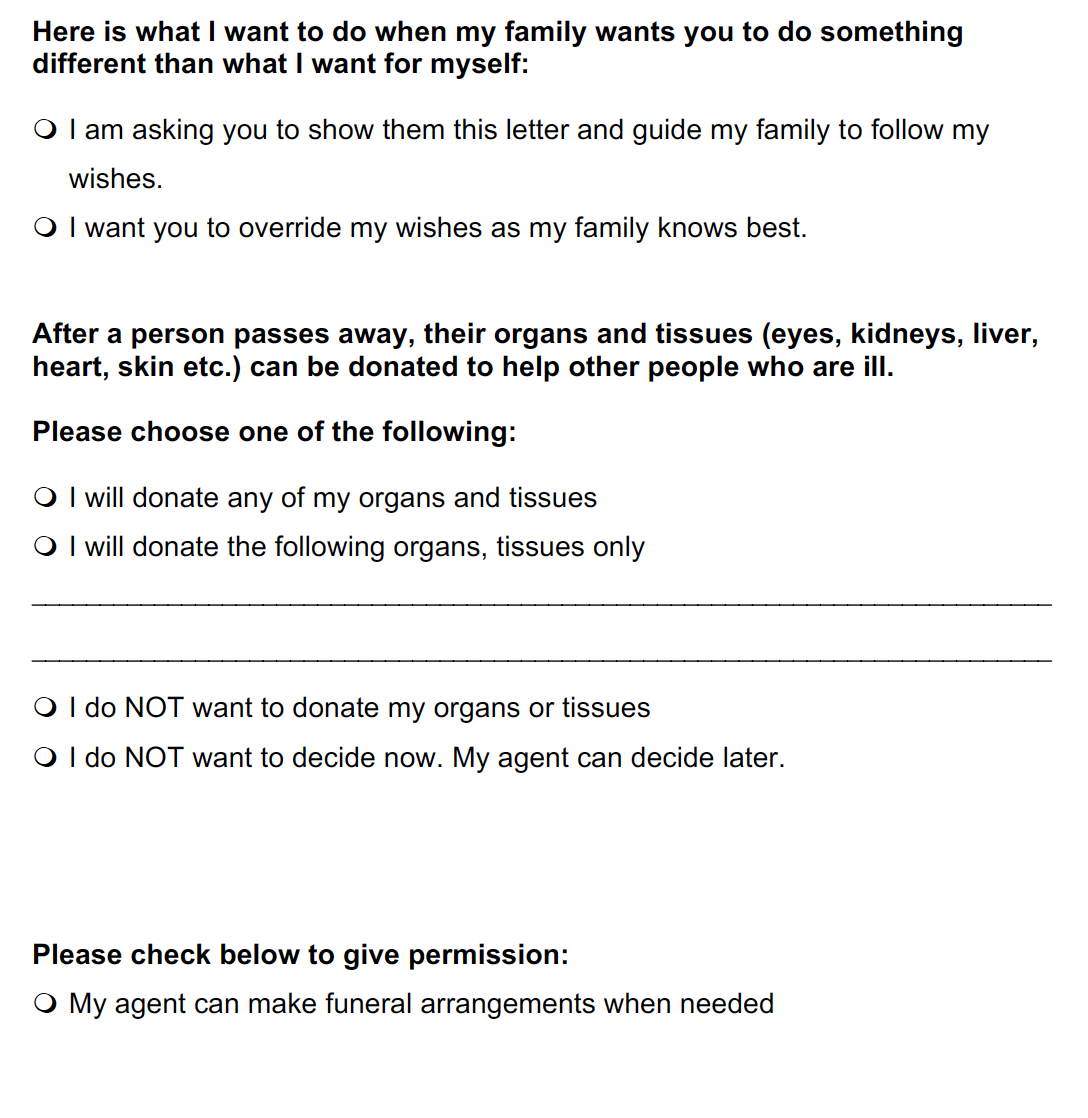


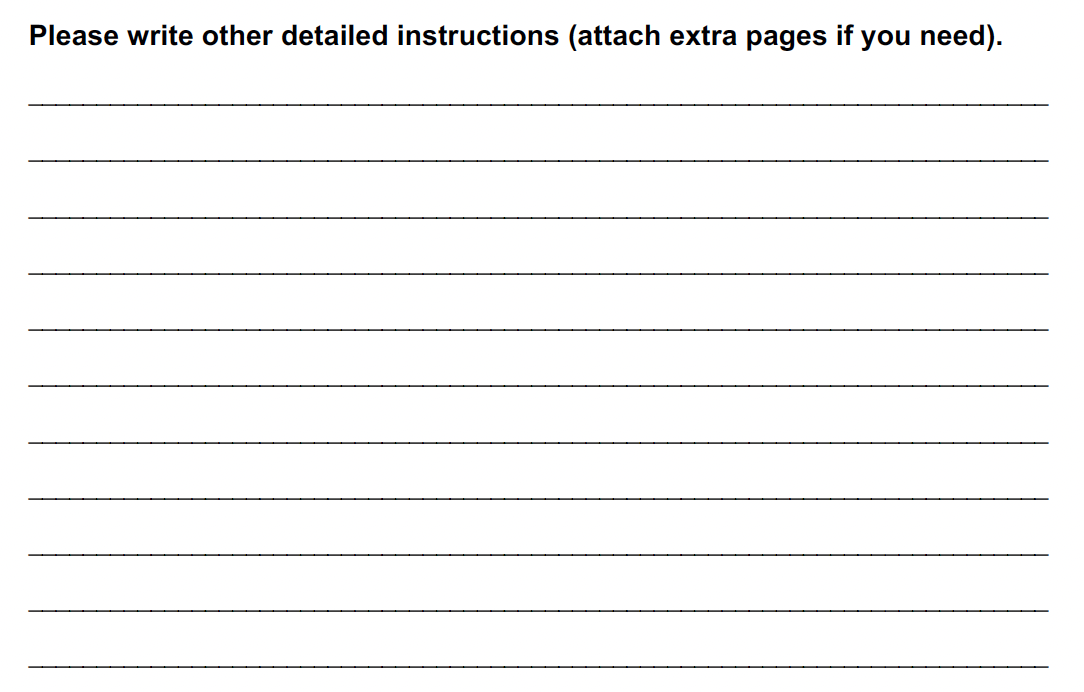


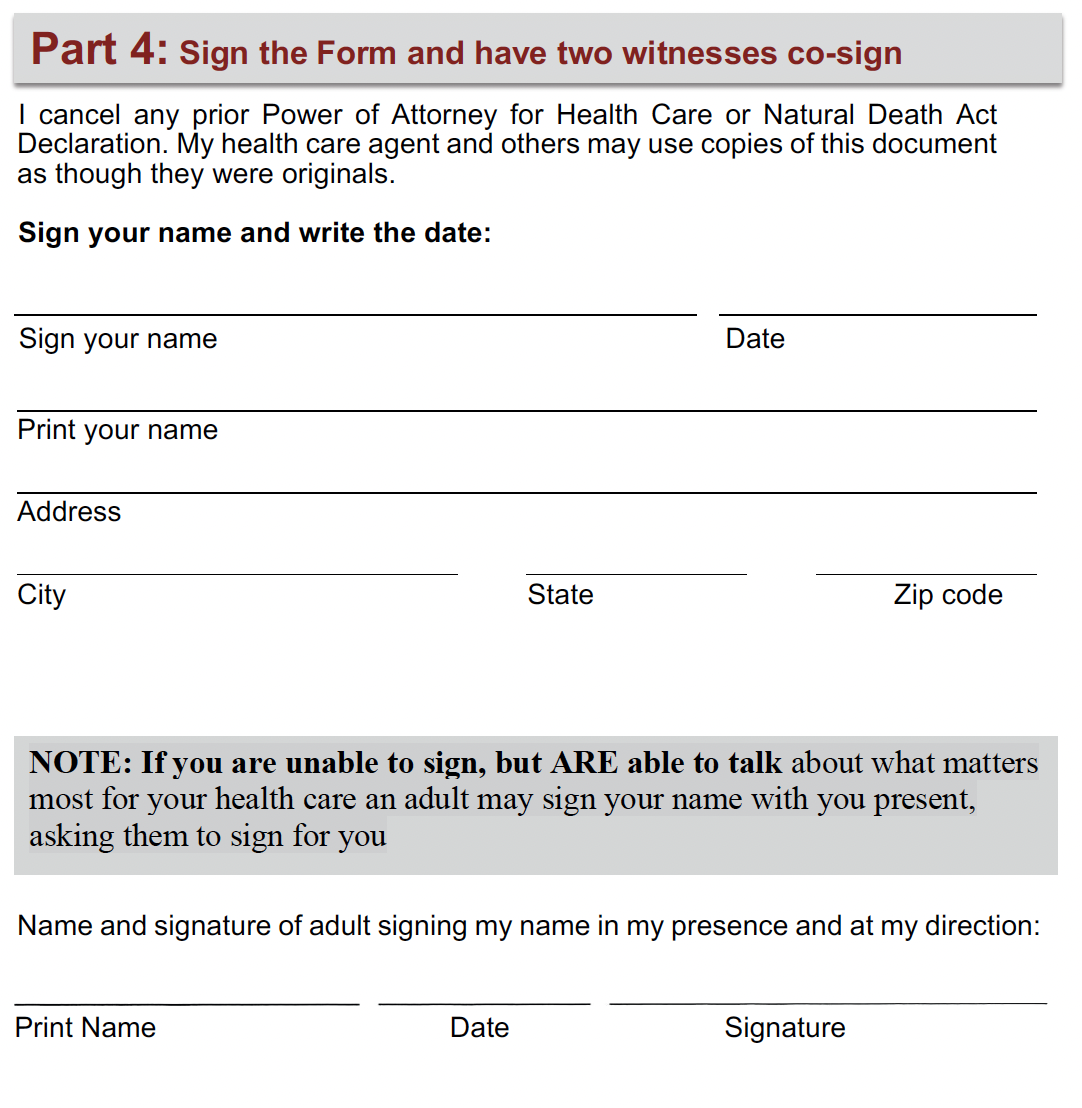


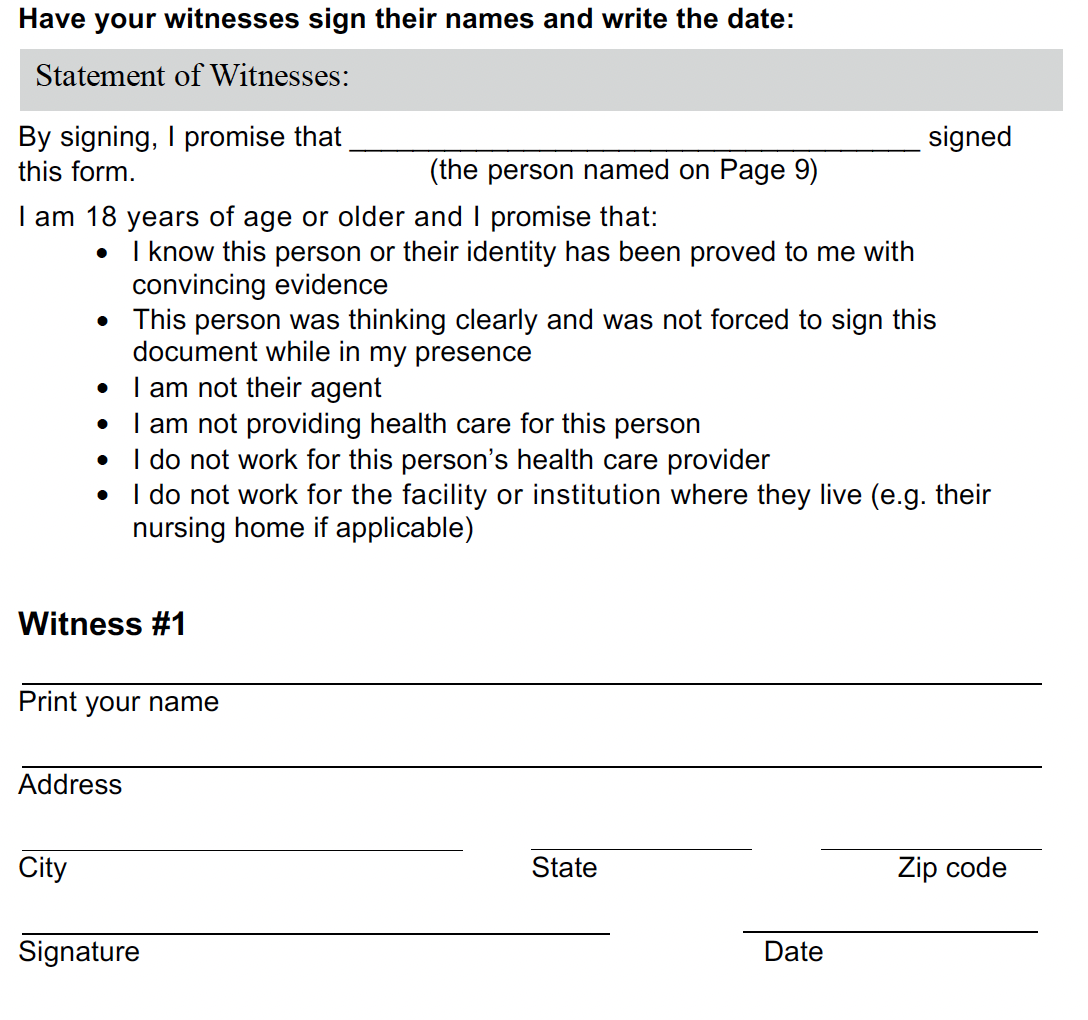


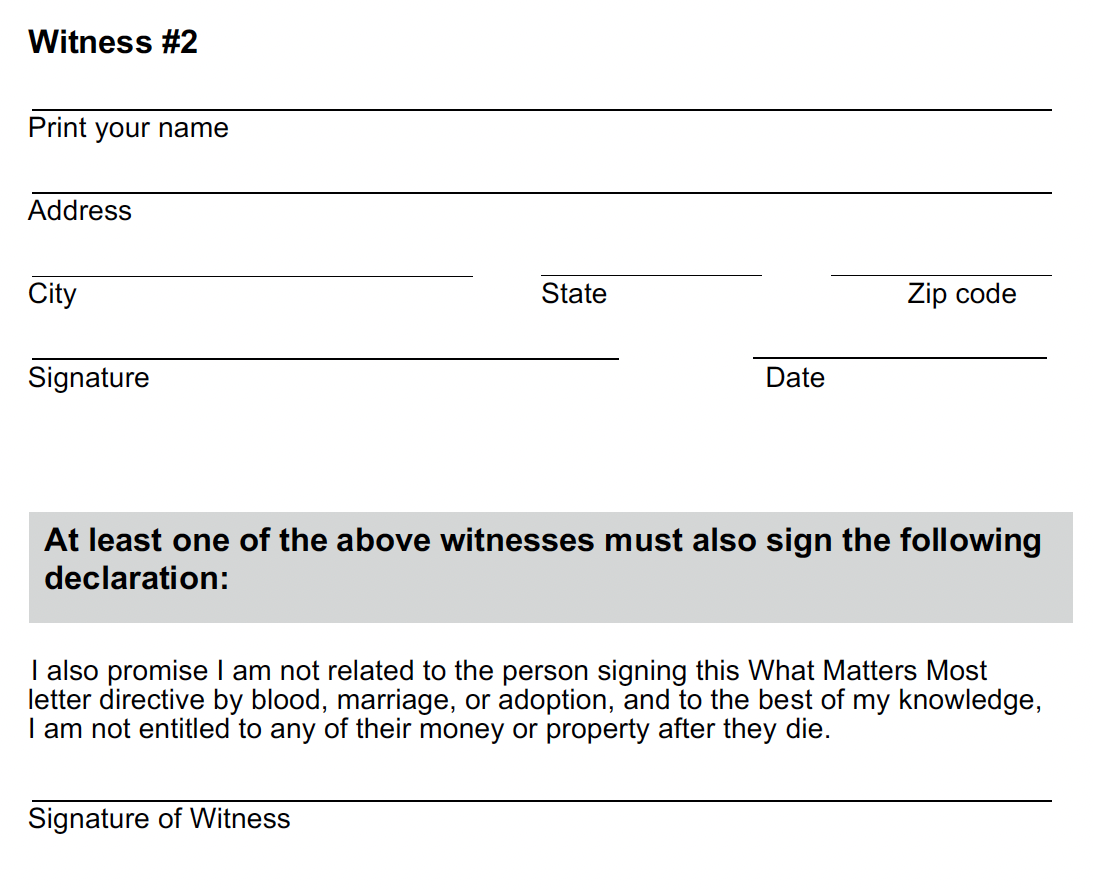


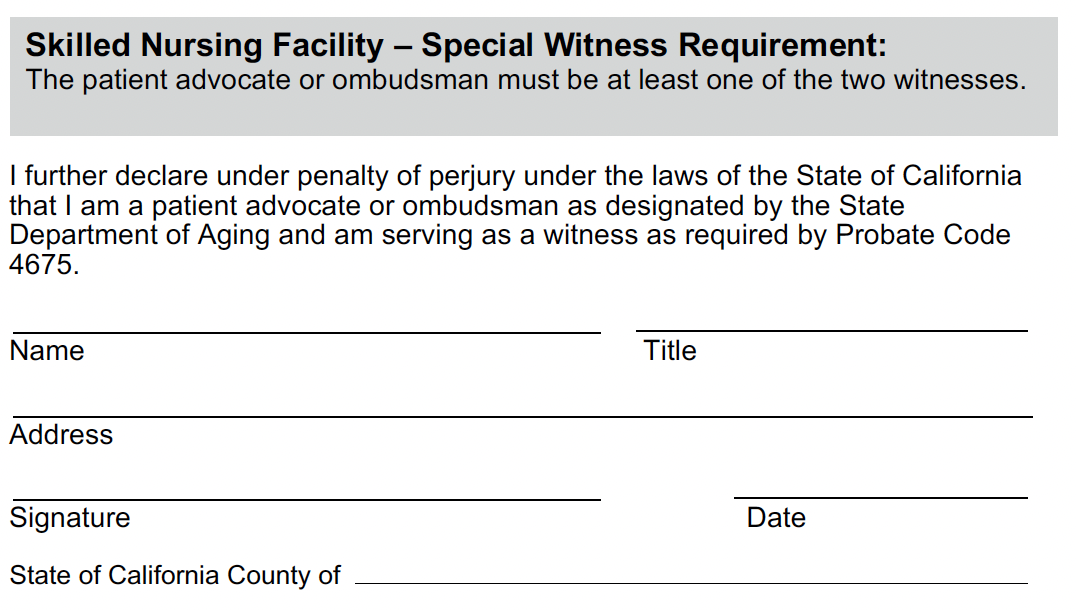


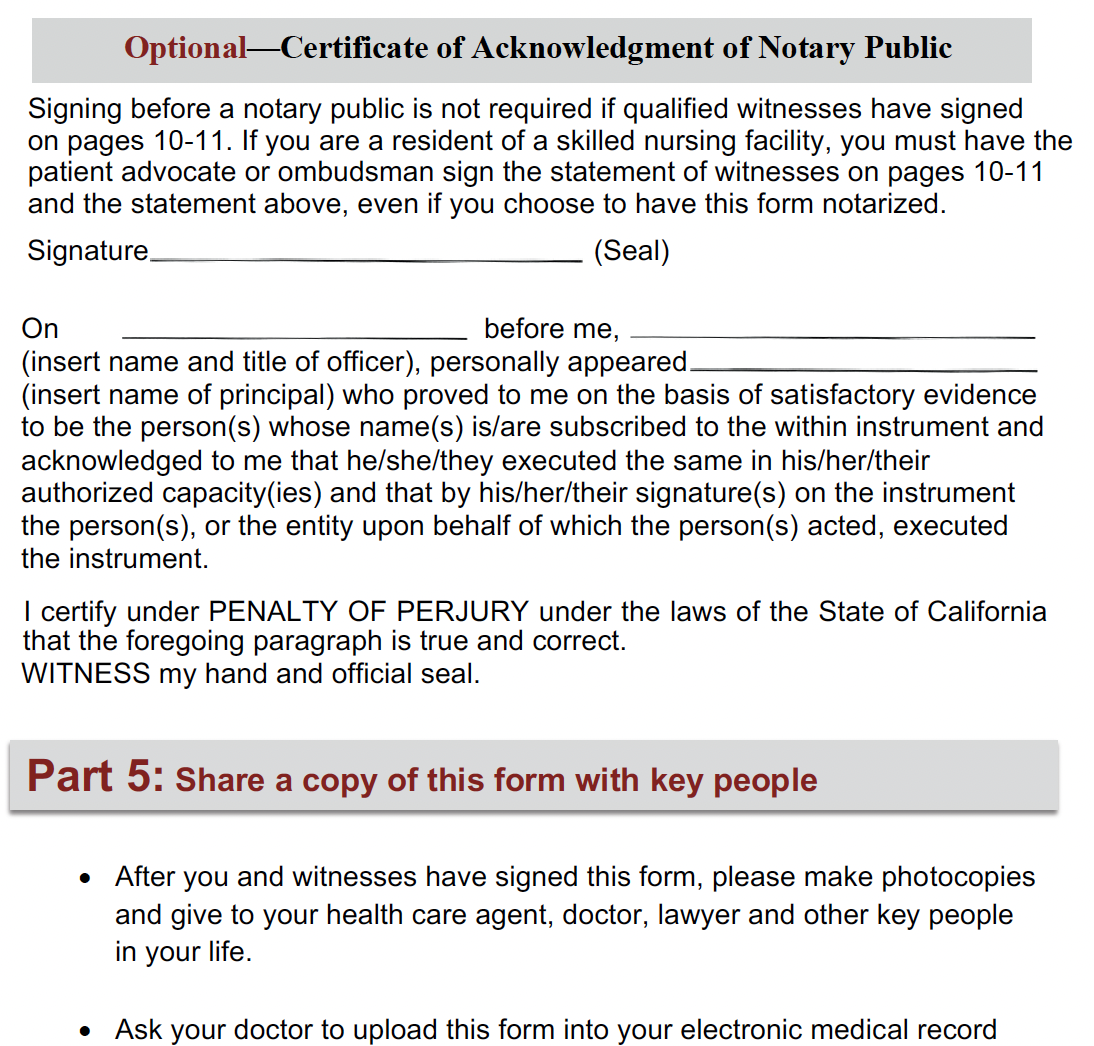


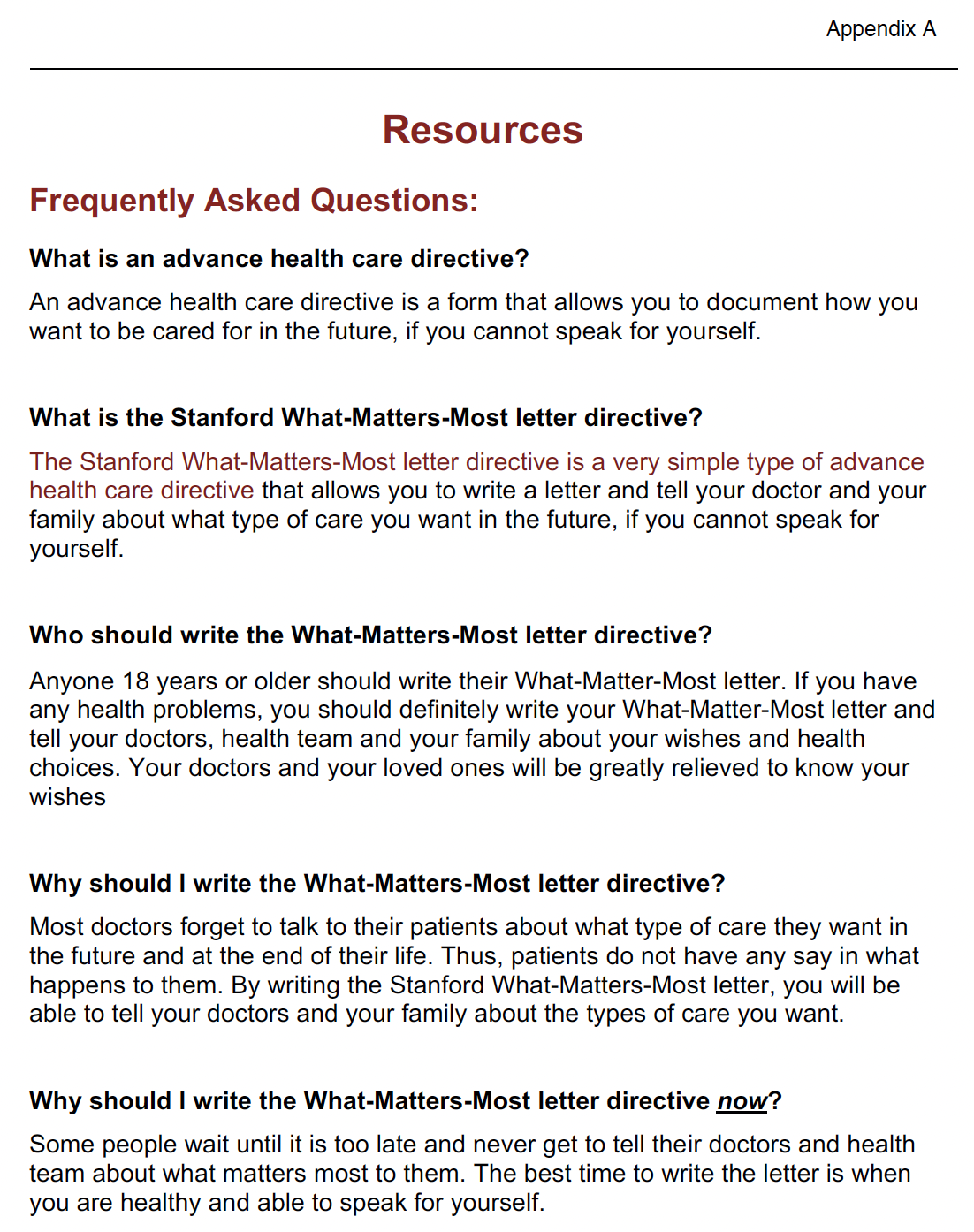


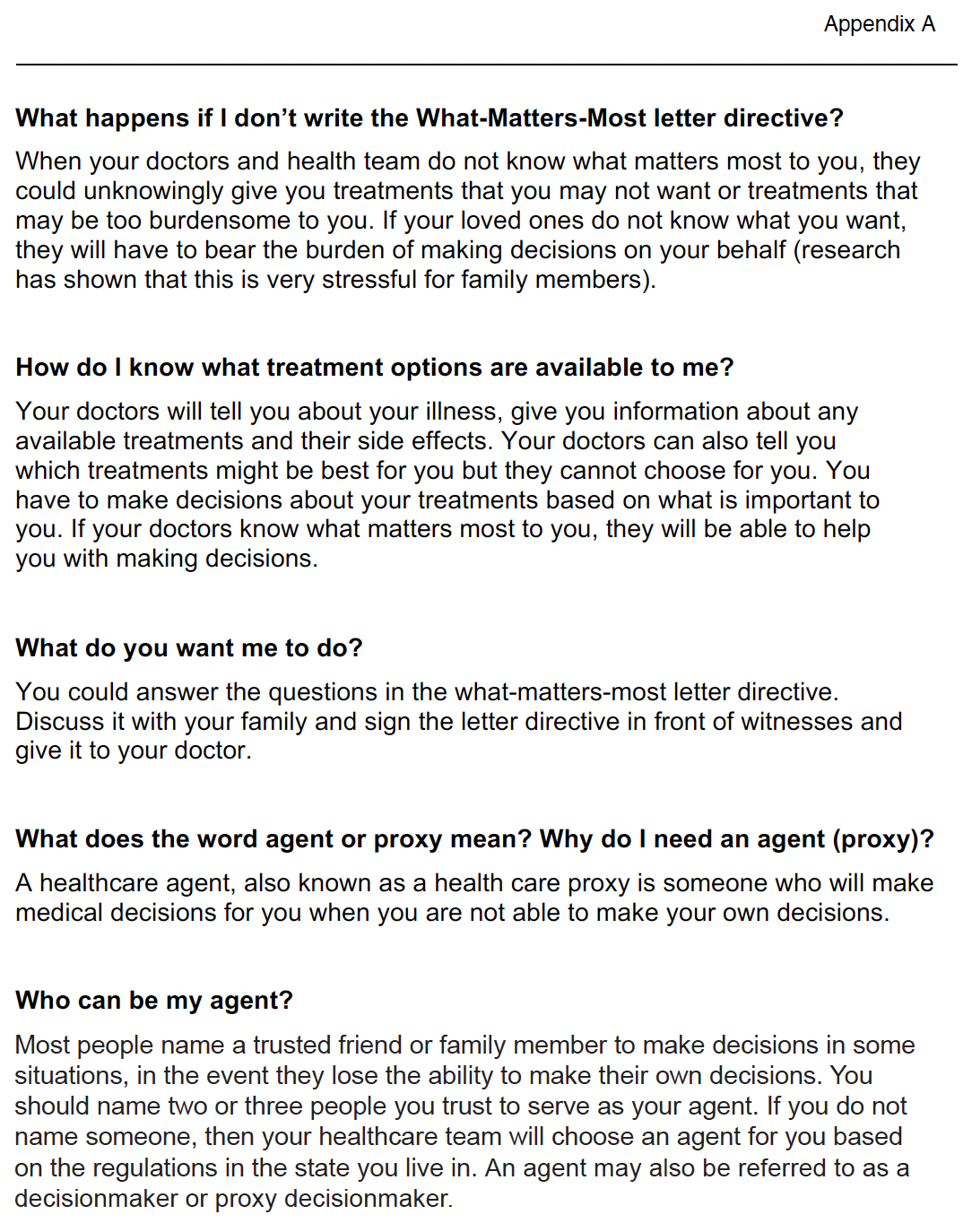


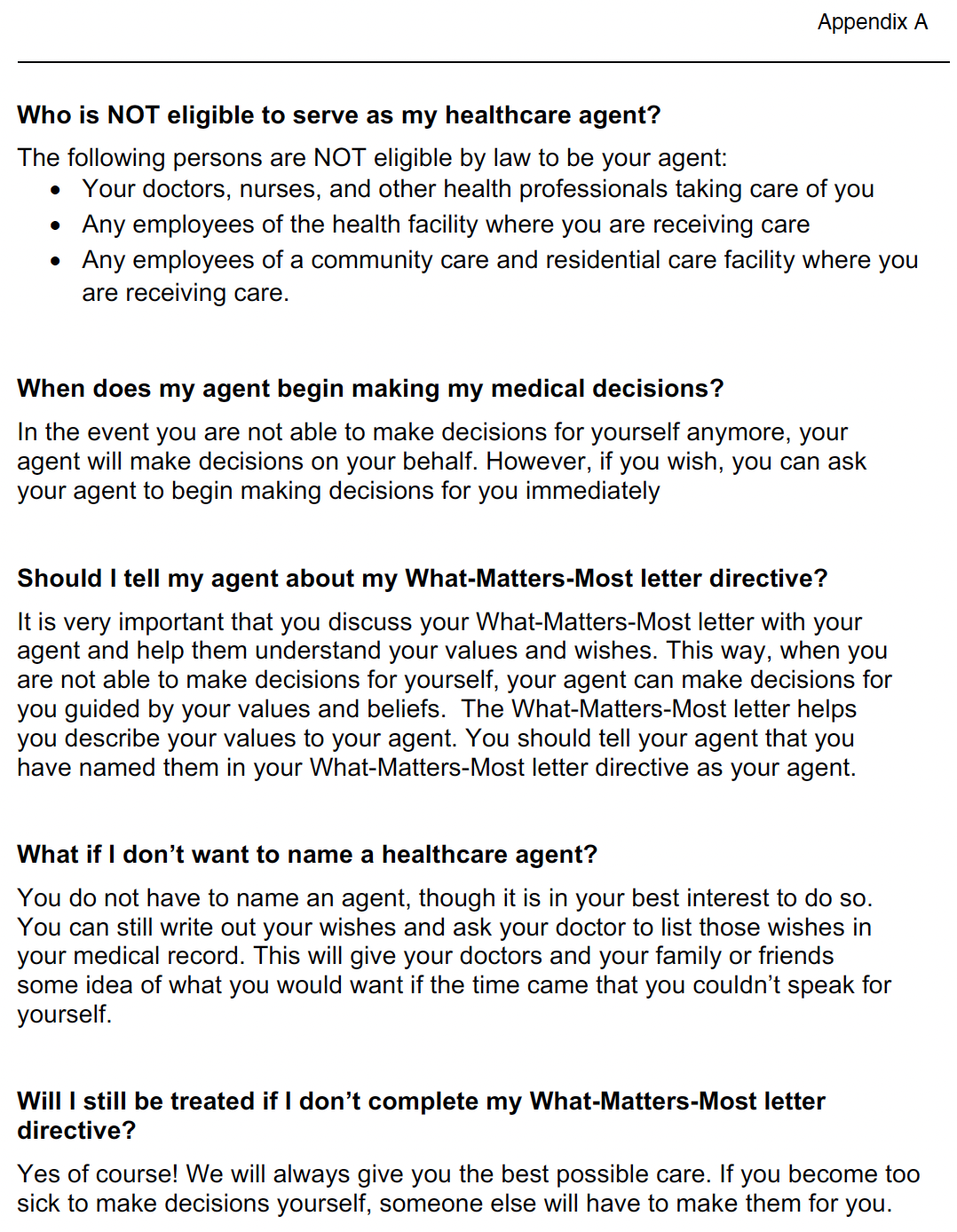


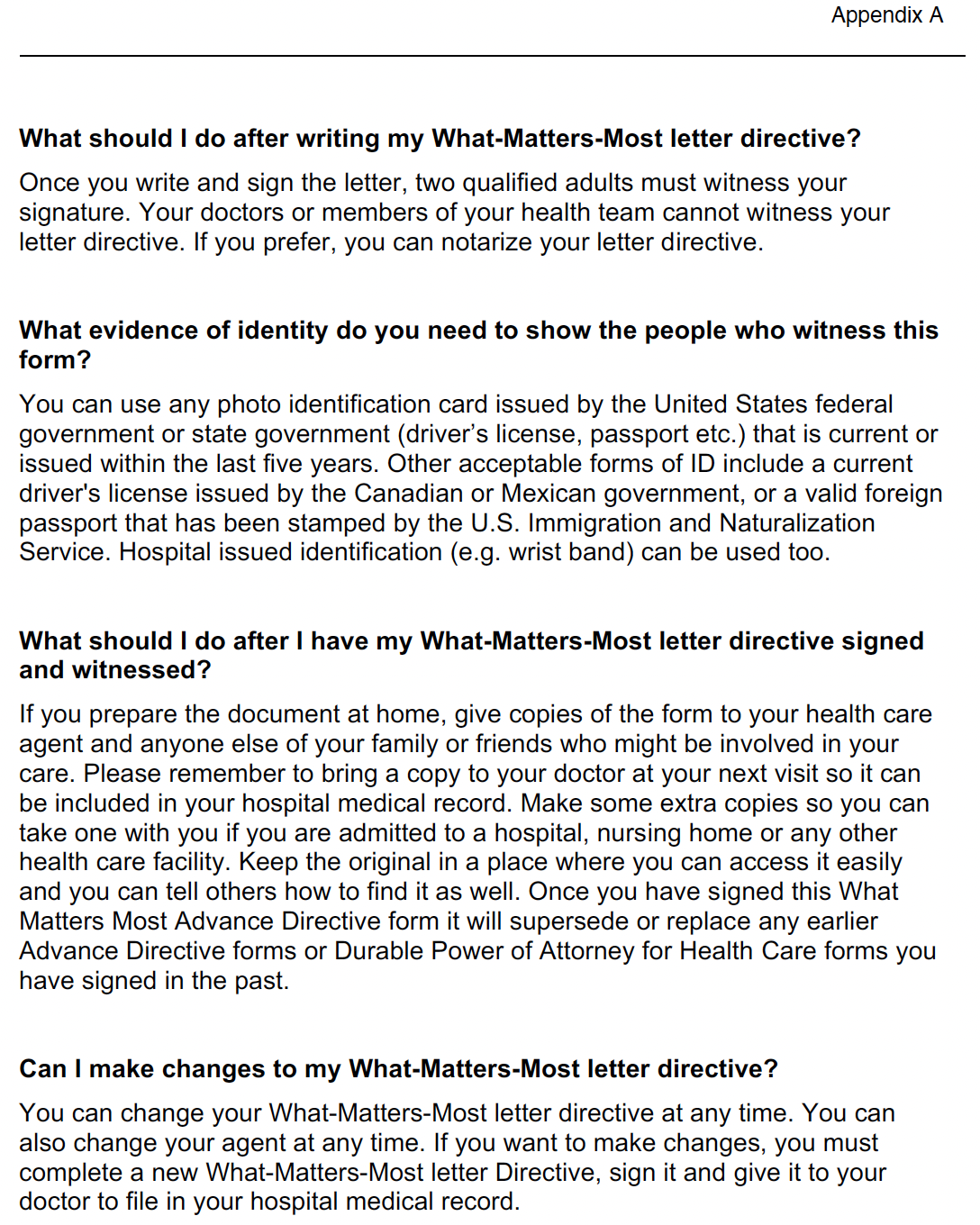


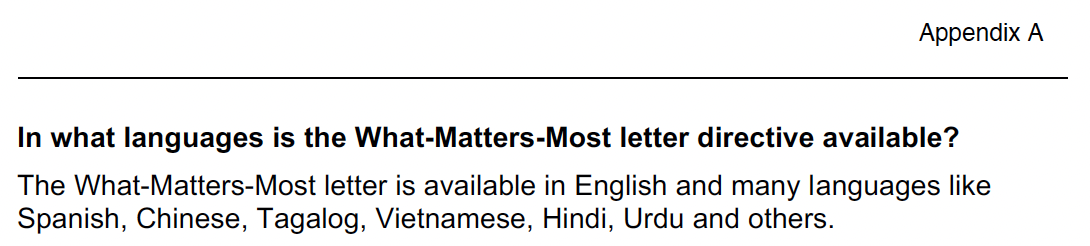


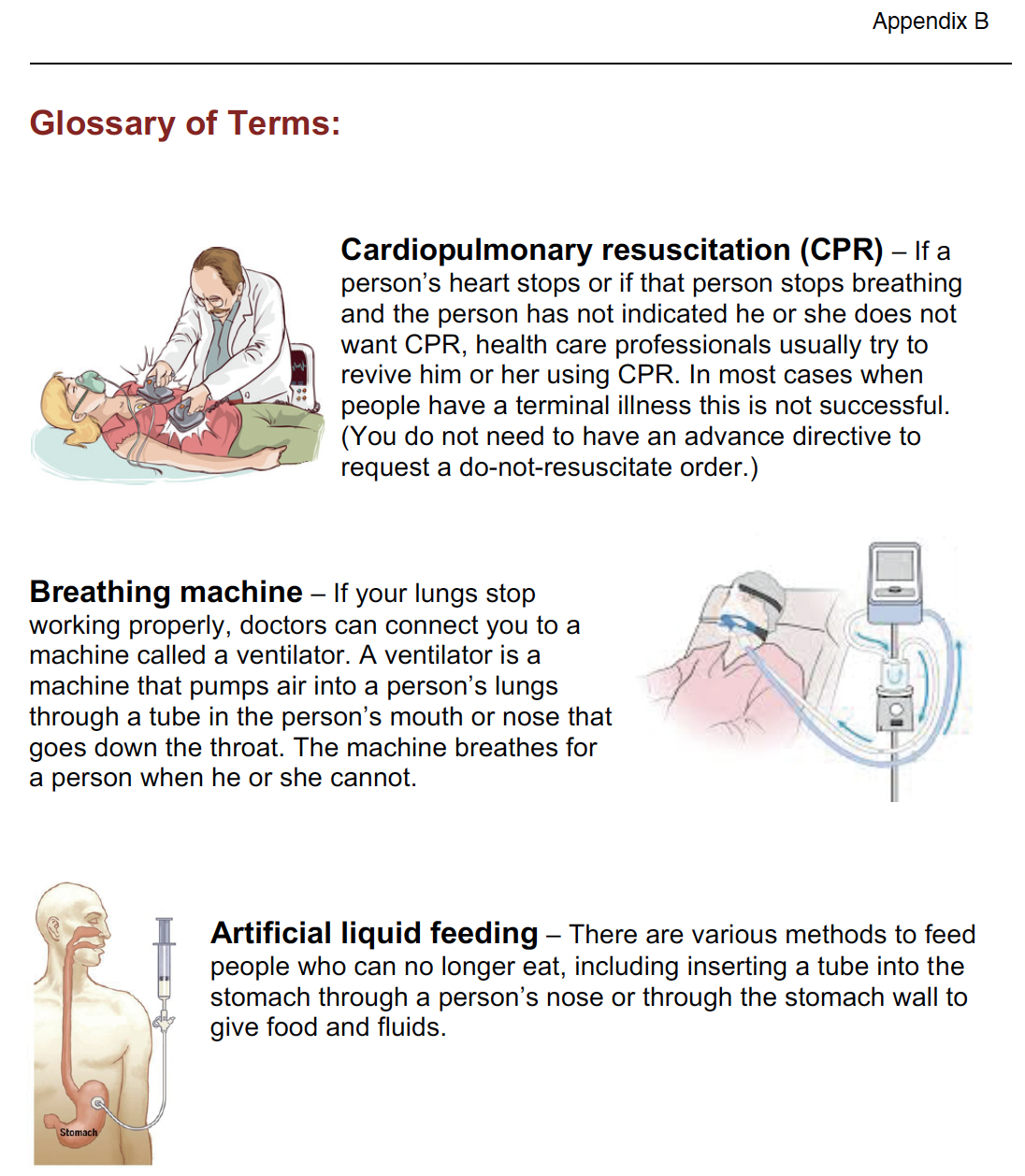


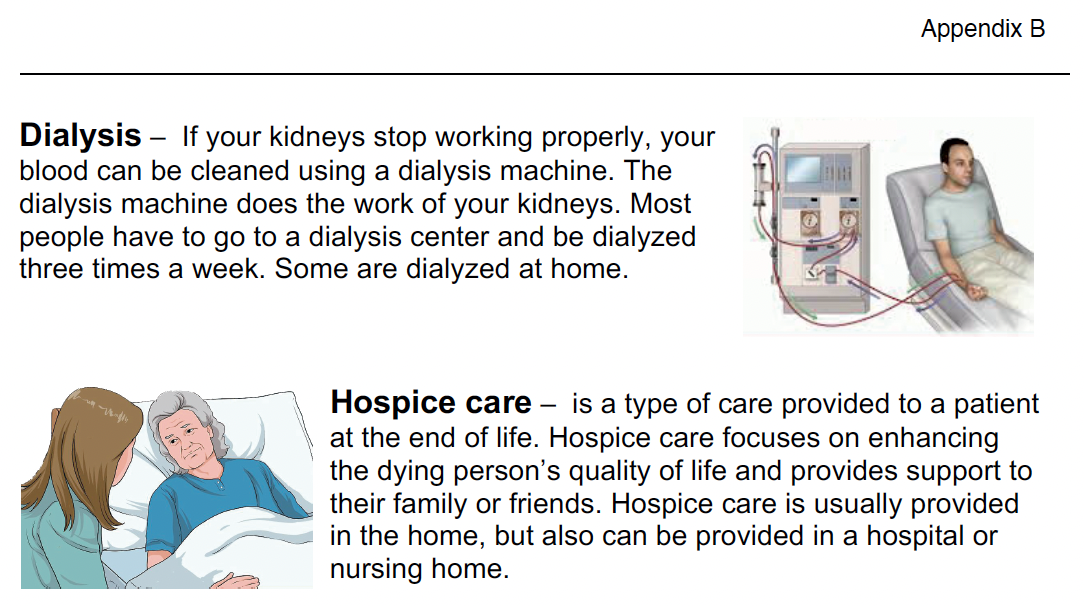


**Physicians Orders for Life Sustaining Treatment (POLST)[7] :** The Physician Orders for Life-Sustaining Treatment (POLST) form empowers seriously ill patients to make their wishes for end-of-life care known and respected. It addresses choices about medical treatments, resuscitation, and interventions such as ventilators or feeding tubes. Printed on bright pink paper, the POLST needs to be signed by both the patient and their primary care provider. The POLST helps prevent unwanted or non-beneficial treatments, minimizes patient and family distress, and ensures that care aligns with the patient’s values and goals.

**EVALUATION MEASURES**

**EVALUATION MEASURES FOR ALL STUDY PARTICIPANTS:** The evaluation measures were implemented by evaluators who were blinded to the treatment condition of the patients. The evaluators met with the patient on study entry (baseline), months 4 and 12 and had them complete the outcome measures described below.

**A. Open ended trigger questions to assess the participants care needs during the study period**

a. How are you doing?

b. Do you need help with anything ? Is there any service or supportive care that we can provide you with?
c. Do you have any concerns, questions or feedback?

*[At 0, 4 months, and one year]*

**B. Longitudinal, observational follow up by blinded evaluators with the goal of tracking all study participants over time**

a. The completion of goals of care conversation and documentation of advance directives and POLST in the electronic health records

*[Ongoing monitoring of the electronic health records during the 12-month intervention period]*

b. Tracking time and place of death of the participants

*[Ongoing monitoring through the electronic health records after completion of the intervention]*

c. Tracking the end-of-life trajectory and the resource utilization -hospital admissions and days, emergency visits in the last six months of life.

*[Ongoing monitoring through the electronic health records after completion of the intervention]*

**Secondary measures:** completed by the evaluators on all study participants at baseline, 4 and 12 months.

| Name | Description | Reference |
| --- | --- | --- |
| ESAS | A tool that helps assess nine common symptoms (pain, tiredness, drowsiness, nausea, lack of appetite, depression, anxiety, shortness of breath, and wellbeing and if any, patient specific symptom). The severity of each symptom is rated from a scale of 0 to 10, where 0 means the symptom is “absent” and 10 means the symptom is “at worst possible severity”. | Nekolaichuk C, Watanabe S, Beaumont C. The Edmonton Symptom Assessment System: a 15-year retrospective review of validation studies (1991--2006). Palliat Med 2008 Mar;22(2):111–122. PMID:18372376 |
| Quality of Life: Alzheimer’s Disease measure(QoL-AD)[9] | This tool is administered in an interview format. The patient rates different aspects of their life using one of four words (poor, fair, good or excellent). | Logsdon RG, Gibbons LE, McCurry SM, Teri L. Quality of Life-Alzheimer’s Disease Scale. Psychosomatic Medicine 1999; Available from: https://psycnet.apa.org/doiLanding?doi=10.1037/t03352-000 [accessed Mar 26, 2025] |
| Patient Activation Measure (PAM) | This tool assesses an individual’s knowledge, skills and confidence in managing their health and healthcare. | Hibbard JH, Stockard J, Mahoney ER, Tusler M. Development of the Patient Activation Measure (PAM): Conceptualizing and Measuring Activation in Patients and Consumers. Health Services Research 2004 Aug;39(4p1):1005–1026. doi: 10.1111/j.1475-6773.2004.00269.x |
| Zarit Burden Interview | This self-report tool assesses the level of burden experienced by caregivers of people with chronic illnesses including those with cognitive impairment. | Zarit SH, Reever KE, Bach-Peterson J. Relatives of the impaired elderly: correlates of feelings of burden. Gerontologist 1980 Dec;20(6):649–655. PMID:7203086 |

References:

1. Ferrell BR, Twaddle ML, Melnick A, Meier DE. National Consensus Project Clinical Practice Guidelines for Quality Palliative Care Guidelines, 4th Edition. Journal of Palliative Medicine Mary Ann Liebert, Inc., publishers; 2018 Dec 1;21(12):1684–1689. doi: 10.1089/jpm.2018.0431

2. Ownby KK. Use of the distress thermometer in clinical practice. Journal of the advanced practitioner in oncology 2019;10(2):175. Available from: https://pmc.ncbi.nlm.nih.gov/articles/PMC6750919/ [accessed Mar 26, 2025]

3. Katz S, Ford AB, Moskowitz RW, Jackson BA, Jaffe MW. Studies of illness in the aged: the index of ADL: a standardized measure of biological and psychosocial function. jama American Medical Association; 1963;185(12):914–919. Available from: https://jamanetwork.com/journals/jama/article-abstract/666768?casa_token=NAKYfmP8kBoAAAAA:NaxfWYwcXPjlH5F_aiEaAYtt6uT6-TXK85zUI_bXMoQ_4Po17uN4x_ITHvd-dV0EiagA-lRfvw [accessed Mar 26, 2025]

4. Sheikh JI, Yesavage JA. Geriatric Depression Scale (GDS): recent evidence and development of a shorter version. Clinical gerontology Routledge; 2014. p. 165–173. Available from: https://www.taylorfrancis.com/chapters/edit/10.4324/9781315826233-11/geriatric-depression-scale-gds-javaid-sheikh-jerome-yesavage [accessed Mar 26, 2025]

5. Periyakoil VS, Neri E, Kraemer H. Common Items on a Bucket List. Journal of Palliative Medicine 2018 May;21(5):652–658. doi: 10.1089/jpm.2017.0512

6. Periyakoil VS, Neri E, Kraemer H. A Randomized Controlled Trial Comparing the Letter Project Advance Directive to Traditional Advance Directive. Journal of Palliative Medicine 2017 Sept;20(9):954–965. doi: 10.1089/jpm.2017.0066

7. Physician Orders for Life-Sustaining Treatment (POLST). Available from: https://capolst.org/wp-content/uploads/2020/10/POLST_2017_wCover.pdf

8. Nekolaichuk C, Watanabe S, Beaumont C. The Edmonton Symptom Assessment System: a 15-year retrospective review of validation studies (1991--2006). Palliat Med 2008 Mar;22(2):111–122. PMID:18372376

9. Logsdon RG, Gibbons LE, McCurry SM, Teri L. Quality of Life-Alzheimer’s Disease Scale. Psychosomatic Medicine 1999; Available from: https://psycnet.apa.org/doiLanding?doi=10.1037/t03352-000 [accessed Mar 26, 2025]

10. Hibbard JH, Stockard J, Mahoney ER, Tusler M. Development of the Patient Activation Measure (PAM): Conceptualizing and Measuring Activation in Patients and Consumers. Health Services Research 2004 Aug;39(4p1):1005–1026. doi: 10.1111/j.1475-6773.2004.00269.x

11. Zarit SH, Reever KE, Bach-Peterson J. Relatives of the impaired elderly: correlates of feelings of burden. Gerontologist 1980 Dec;20(6):649–655. PMID:7203086
